# A manually curated gene–phenotype catalogue for progeroid syndromes and premature aging

**DOI:** 10.18632/aging.206366

**Published:** 2026-03-30

**Authors:** Nuša Likar, Tanja Kunej

**Affiliations:** 1 University of Ljubljana, Biotechnical Faculty, Department of Animal Science, 1230 Domžale, Slovenia

**Keywords:** aging, premature aging, progeroid syndromes, DNA repair, *LMNA* gene

## Abstract

Progeroid syndromes (PS) are a heterogeneous group of rare hereditary disorders with features resembling premature aging, thereby serving as valuable models for studying human aging biology. However, data on these syndromes remain fragmented across literature sources, with inconsistent terminology and classifications hindering systematic analyses. To address these challenges, we developed a curated catalogue integrating information from 84 publications and the Online Mendelian Inheritance in Man (OMIM) database. This resource consolidates data on 144 genes linked to 56 syndromes and their subtypes, comprising 160 distinct clinical entities, and their associated clinical manifestations categorized into 18 clinical feature groups. The compiled data were visualized and analyzed through a genome–phenome association network, offering new insights into the genetic and phenotypic heterogeneity of these disorders. The gene set was further analyzed through a protein–protein interaction (PPI) network and functional enrichment analysis, revealing a highly interconnected protein network with pronounced enrichment of genome maintenance pathways. Ten highly connected hub genes were prioritized in the PPI network based on degree centrality and further examined in the context of aging by cross-referencing with the Open Genes database, a curated resource of human genes associated with aging and longevity. A case study of the *LMNA* gene illustrated the pleiotropic impact of single-gene variants across multiple syndromes and related disorders beyond classical PS. Overall, this study provides a reference resource and framework to support future research into premature aging syndromes and their broader implications for understanding physiological aging.

## INTRODUCTION

Aging is an inevitable process that affects all human beings and is characterized by a gradual decline in cellular and tissue functions, resulting in an increased vulnerability to disease and death [1–7]. This decline involves various molecular, cellular, and systemic changes, such as genomic instability, telomere attrition, epigenetic alterations, loss of proteostasis, disabled macroautophagy, deregulated nutrient-sensing, mitochondrial dysfunction, cellular senescence, stem cell exhaustion, altered intercellular communication, chronic inflammation, and dysbiosis, collectively referred to as the “hallmarks of aging” [1, 8]. Despite significant advances in understanding these processes, the complexity of aging remains incompletely understood, largely due to the ethical, practical, and temporal challenges inherent in studying human aging, which unfolds over decades [3, 9]. As a result, short-lived model organisms are often used to study the molecular mechanisms of aging, but translating such findings to humans can often be limited [9, 10]. An alternative strategy to deepen our understanding of aging mechanisms involves studying rare human disorders where aging-like processes are accelerated, paralleling natural aging [3, 9–13].

Human progeroid syndromes, also referred to as premature aging disorders, are a heterogeneous group of rare hereditary diseases characterized by clinical features that resemble aspects of accelerated aging [2, 4, 6, 11, 14, 15]. Premature aging signs include, among others, alopecia, hair graying, vision and hearing loss, osteoarthritis and osteoporosis, cardiovascular disease, lipodystrophy, loss of muscle mass, skin atrophy, progressive neurodegeneration, type 2 diabetes mellitus, and various malignancies [3, 9, 11, 12, 14–16]. These features become evident at earlier ages and often progress rapidly, leading to shortened lifespans [10, 11, 15, 17]. Depending on the specific syndrome, disease onset can range from congenital or neonatal and early childhood forms to juvenile or early adulthood-onset presentations, with aging-like manifestations emerging decades earlier than in physiological aging [10, 12, 14, 15]. In addition to typical aging phenotypes, developmental delays, intellectual disabilities, and growth retardation as well as bone deformities and facial abnormalities typical of the disease may also occur [12, 15]. Although no single progeroid syndrome reproduces the entirety of physiological aging, they frequently accelerate multiple aging-associated traits, earning the designation “segmental progeroid syndromes” [5, 12, 15–18]. Clinically, these syndromes are highly heterogeneous: they present with distinct sets of phenotypes, affect different tissues and organs, and vary in age of onset, disease course, and severity; on the other hand, they also exhibit many overlapping phenotypes, which complicate accurate diagnosis [12, 14, 16, 19].

The etiological mechanisms underlying progeroid syndromes equally exhibit considerable diversity and depend on the affected gene and the resultant perturbation in cellular pathways [11]. Most are monogenic disorders in which single-gene variants lead to malfunctions that impact, with varying fidelity, multiple aspects of the senescent phenotype [5, 10, 17, 20]. Their monogenic nature makes them relatively straightforward to investigate, enabling the exploration of the underlying causes of their pathology, the identification of links between specific genetic variants and aging phenotypes, and insights into general processes and pathways that also play a role in physiological aging [5, 10]. This, in turn, opens avenues for the identification of shared biomarkers and the exploration of therapeutic strategies, as demonstrated in recent studies employing progeroid disease models [21, 22]. Notably, variants in these same genes are also implicated in a spectrum of other diseases that extend beyond classical progeroid syndromes. A quintessential example is the *LMNA* gene, variants in which cause Hutchinson-Gilford Progeria Syndrome (HGPS), the most extensively studied among premature aging disorders. Variants in the *LMNA* gene have been linked to a variety of human disorders, collectively known as primary laminopathies, including not only premature aging syndromes but also lipodystrophies, neuropathies, cardiomyopathies, and muscular dystrophies with partially overlapping phenotypes [14, 23–27].

Thus far, more than 100 syndromes associated with signs of premature aging have been described [12, 14, 15]. However, due to the vast scope of the field, data on these syndromes are scattered across multiple publications and databases, including curated resources such as the Online Mendelian Inheritance in Man (OMIM), and the terminology and categorization of genes and diseases are not fully standardized, making knowledge synthesis and systematic analysis challenging.

The aims of this study were therefore to: (1) develop a manually curated catalogue on progeroid syndromes, compiling and systematically organizing genetic factors, associated syndromes, their subtypes, and clinical features, along with standardized gene terminology and applied categorizations; (2) visualize and analyze the compiled data through a genome–phenome association network; (3) utilize the compiled gene list to construct a protein–protein interaction (PPI) network, identify enriched biological pathways and processes, and prioritize highly connected hub genes; and (4) illustrate the phenotypic diversity arising from single-gene variants through a network of *LMNA*-associated diseases and their clinical manifestations. With these approaches, we aimed to consolidate previously reported information into a unified and accessible framework that would support improved classification, diagnosis, and future research into premature aging syndromes and their broader implications for understanding physiological aging, while also providing a transferable methodological model for integrating, visualizing, and analyzing genome–phenome data in other complex disorders.

## RESULTS

**Figure 1:**
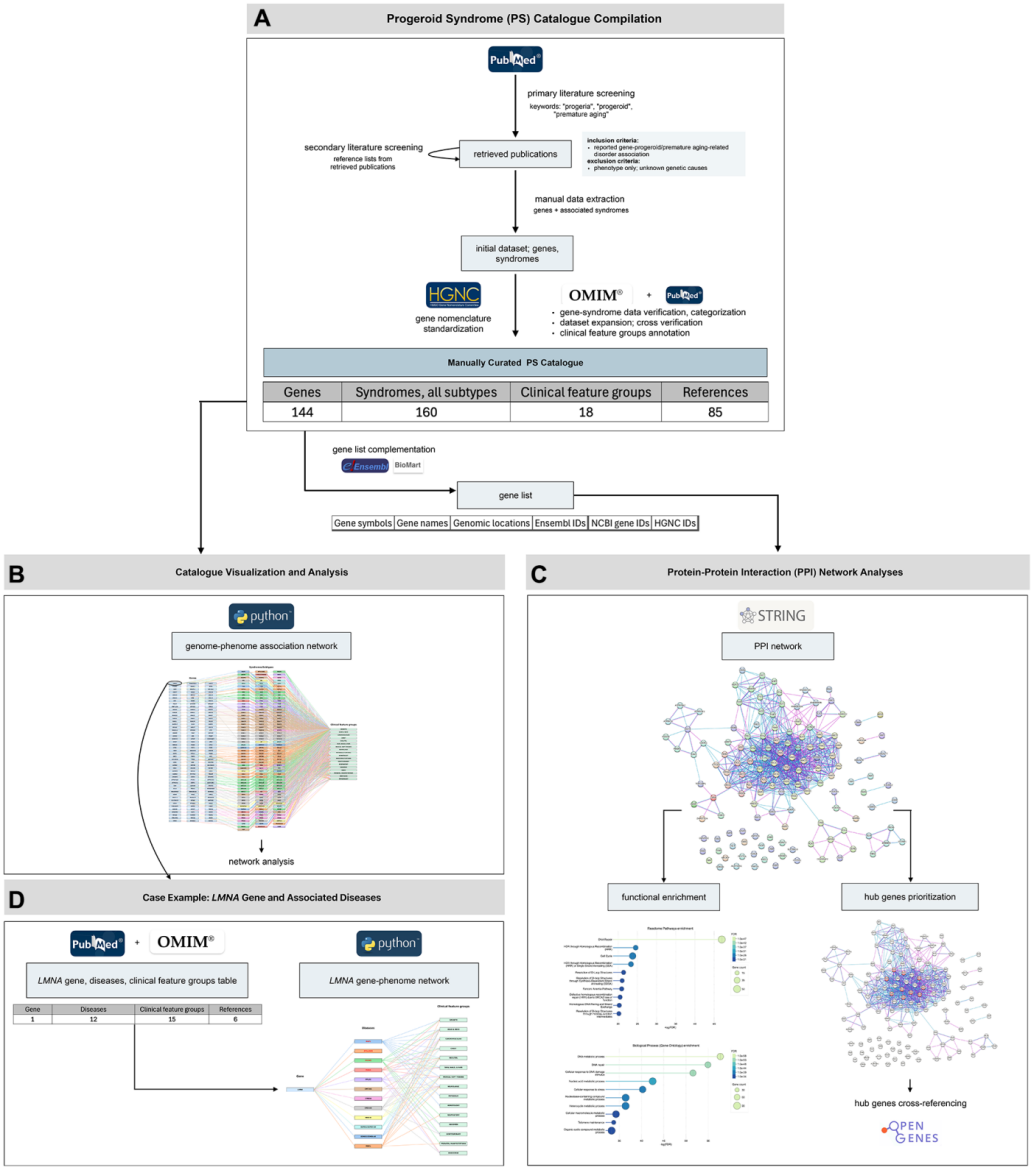
**Overview of the study workflow and main results.** The study proceeded through four major stages. (**A**) Progeroid syndrome (PS) catalogue compilation: We generated a manually curated PS catalogue following a structured workflow of primary and secondary literature screening, manual data extraction, data verification and categorization, dataset expansion, gene nomenclature standardization, and clinical feature groups annotation. The final dataset includes 144 genes, associated with a total of 160 clinical entities and 18 clinical feature groups, compiled from 84 publications and the OMIM database. (**B**) Catalogue visualization and analysis: The curated dataset was visualized through a genome–phenome association network and further explored though network analysis to assess genetic and phenotypic heterogeneity. (**C**) Protein–protein interaction (PPI) network analyses and functional characterization: The compiled gene set was analyzed through PPI network construction, functional enrichment of biological processes and pathways, and prioritization of highly connected hub genes, followed by cross-referencing with the Open Genes database. (**D**) Case example – *LMNA* gene: To illustrate single-gene pleiotropy, *LMNA*-associated diseases and their clinical feature groups were summarized in a dedicated table and a gene–phenotype network.

Through the integration of data from published literature and the OMIM database, we created a manually curated resource on progeroid and related premature aging disorders, encompassing the syndromes, their subtypes, associated genes, and clinical feature groups categorized by affected organ systems or body regions. The compiled data were visualized through a genome–phenome association network, and network analyses were performed to explore the genetic and phenotypic heterogeneity of these disorders. The compiled gene set was further analyzed through a PPI network and functional enrichment analyses. Ten highly connected hub genes were prioritized within the PPI network and cross-referenced with the Open Genes database, a curated resource of human genes associated with aging and longevity, to evaluate prior links to aging-related processes and phenotypes beyond rare progeroid syndromes. Additionally, we illustrated the phenotypic diversity arising from single-gene variants in the compiled genes through a table and a network of *LMNA*-associated diseases and their clinical manifestations. The study workflow and key results are summarized in Figure 1.

### Overview of the curated progeroid syndrome catalogue (PS catalogue)

The constructed PS catalogue provides an overview of the genes, associated syndromes, their subtypes, and the corresponding clinical manifestations (Supplementary Table 1). The data in the catalogue are organized into six columns: (1) “Gene(s)”: approved HGNC symbols and names of the genes linked to progeroid and related premature aging syndromes, together with their previous and/or alias symbols used in the cited publications; (2) “Syndrome”: abbreviations and names of the syndromes associated with the genes in the first column; (3) “Subtype(s)”: subtypes (abbreviations and names) in cases of syndromes with subtypes (“/” in cases of syndromes without subtypes); (4) “Clinical feature groups”: related groups of clinical signs and symptoms, defined according to the organ systems or body parts affected, as listed in the OMIM database; (5) “Notes”: additional information and clarifications where needed; and (6) “References”: literature and database sources supporting the association of individual genes with the recorded syndromes; at least two supporting references per gene–syndrome association were documented. For greater clarity, white and gray fields alternate in the table; all rows shaded in the same color correspond to the same syndrome but are split according to genes and their respective subtypes, as each subtype is associated with a different gene and distinct clinical feature groups, necessitating separate rows within the same color section.

The dataset includes a total of 144 genes and 56 syndromes, 18 of which have subtypes, totaling 160 distinct clinical entities (with each subtype counted as a separate clinical entity). Among the 144 genes, 142 are protein-coding, while two are non-protein-coding: *TERC*, a long non-coding RNA (lncRNA), and *RNU7-1*, a small nuclear RNA (snRNA), classified as a small RNA (sRNA).

The gene–syndrome association data are compiled from the OMIM database, accessed between December 2024 and February 2025, and 84 publications published between January 2000 and December 2024 [3–6, 10–20, 28–96]. The dataset incorporates both well-established, extensively studied progeroid syndromes as well as newly identified or recently proposed disorders exhibiting features of premature or accelerated aging. During catalogue construction, differences between published sources and database entries were addressed through manual curation. Gene nomenclature was standardized according to HUGO Gene Nomenclature Committee (HGNC) guidelines to ensure consistent terminology and comparability across sources, while previous and/or alias gene symbols were retained to maintain traceability to earlier literature. Syndrome and subtype classification primarily followed the OMIM database as a reference framework. Gene–syndrome associations that showed differences between sources or were not listed in OMIM were nevertheless included if documented in at least two other supporting literature sources; “NA” in the catalogue indicates that subtype designation and/or corresponding clinical feature data could not be assigned based on the available OMIM annotations at the time of curation, while additional clarifications and specifics are provided in the “Notes” column of the catalogue.

The clinical features of the included syndromes are categorized into a total of 18 distinct clinical feature groups, defined according to the organ systems or body parts affected, following the classification provided by the OMIM database. These categories include: growth; head and neck; cardiovascular; chest; skeletal; skin, nails and hair; muscle, soft tissues; neurologic; metabolic features; hematology; endocrine features; genitourinary; respiratory; abdomen; voice; prenatal manifestations; neoplasia; and immunology. This organization facilitates cross-comparison of syndromes and highlights their systemic impact.

Additionally, a separate gene list of the 144 genes associated with the included syndromes (Supplementary Table 2) was constructed, containing their approved symbols and names along with their genomic locations, cytogenetic bands, and the corresponding identifiers (IDs) in the Ensembl, NCBI, and HGNC databases. The genes are sorted by genomic locations, from chromosome 1 to chromosome X.

### Insights from the genome–phenome association network

**Figure 2:**
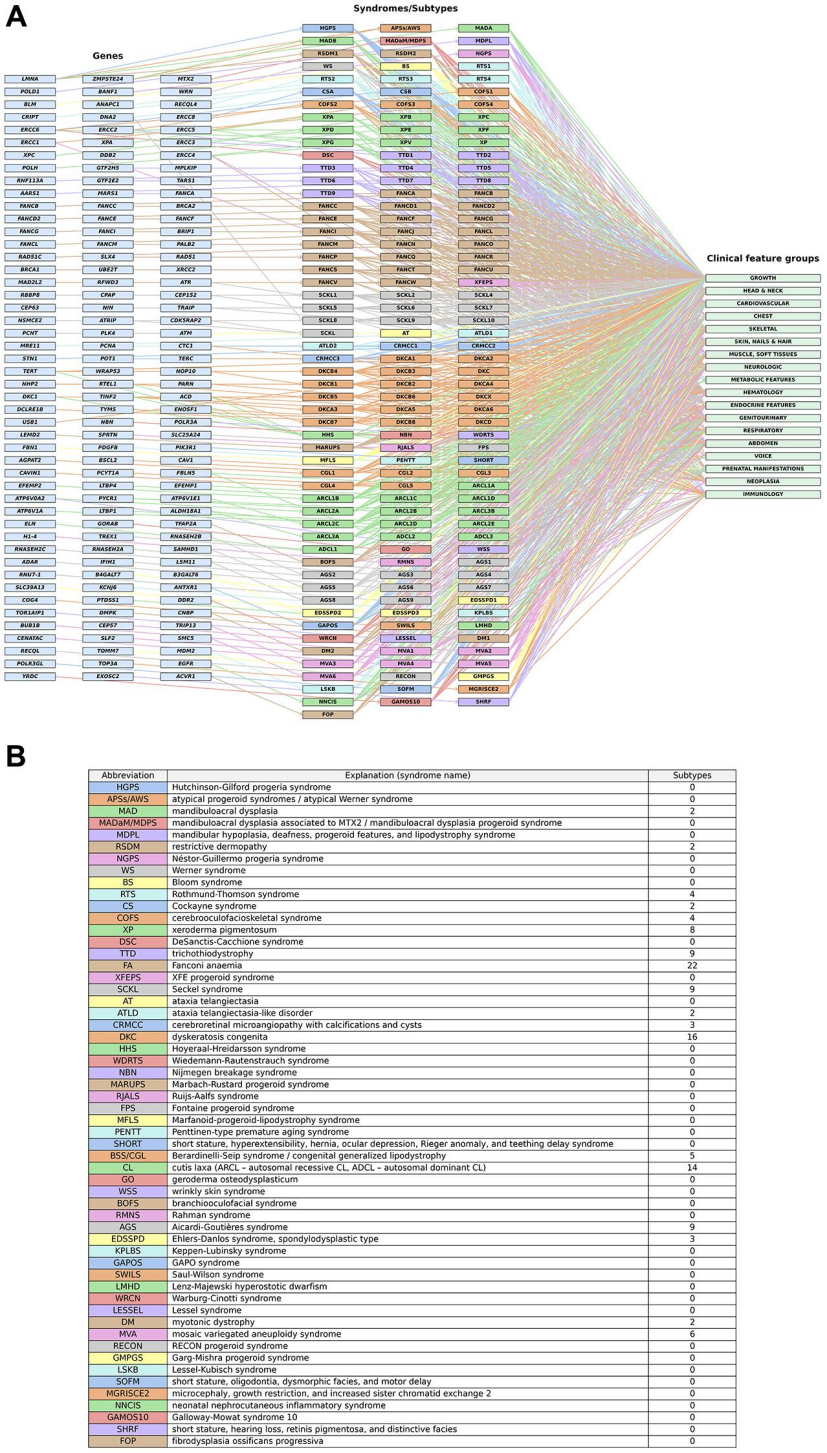
**A genome–phenome view of premature aging syndromes.** (**A**) Each gene (left) is connected to its associated syndrome(s) or subtype(s) (middle), which are further linked to their corresponding clinical feature groups (right). For syndromes with subtypes, a consistent color scheme is applied to indicate grouping, with additional markers (letters or numbers) identifying individual subtypes (e.g., XP: xeroderma pigmentosum; XPA: xeroderma pigmentosum, complementation group A/SCKL: Seckel syndrome; SCKL1: Seckel syndrome 1). (**B**) Explanations of syndrome abbreviations (full syndrome names) and the numbers of subtypes within each syndrome. Details on the syndromes and subtypes are listed in Supplementary Table 1.

Visualization of the compiled dataset (Figure 2) provides an integrative genome–phenome view of the compiled syndromes. Figure 2A depicts 144 genes associated with 56 premature aging syndromes – a total of 160 distinct clinical entities including subtypes – along with their corresponding clinical feature groups. Each gene is connected to its associated clinical entity (syndrome or syndrome subtype), which is then linked to the associated clinical feature groups. Subtypes of a given syndrome are displayed in the same color, while unique identifiers – such as letters or numbers – distinguish between subtypes within the same color group, in accordance with subtype labels from the OMIM database. The legend accompanying the network (Figure 2B) includes explanations of syndrome abbreviations (full syndrome names) and the number of subtypes for each syndrome. Figure 2 is intended to provide a conceptual overview of the structure and scope of the curated dataset, illustrating both the broad scope and heterogeneity among premature aging-related syndromes and their clinical features, as well as the wide array of genetic factors contributing to these disorders. It also served as a foundation for the downstream network analyses used to derive quantitative insights into the genetic and phenotypic heterogeneity of the compiled syndromes.

The network analysis revealed that 19 out of 144 compiled genes are associated with more than one syndrome or syndrome subtype in the dataset. Specifically, the *LMNA* gene is linked to four distinct syndromes: HGPS, APSs/AWS, MAD, and RSDM. Similarly, *ERCC6* is associated with four syndromes: CS, XP, COFS, and DSC; COFS is also considered a severe form of CS, and DSC a more severe clinical phenotype of XP. The genes *ERCC2*, *ERCC4*, and *ZMPSTE24* are each associated with three distinct syndromes in the dataset. Specifically, *ERCC2* is associated with XP, COFS, and TTD, as well as rare combinations of XP with CS (XP/CS complex) and XP with TTD (XP/TTD complex), while *ERCC4* is linked to XP, FA, and XFEPS, and also to XP/CS complex. *ZMPSTE24* is associated with APSs/AWS, MAD, and RSDM. The genes *ERCC3, ERCC5* and *ERCC1* are each associated with two distinct syndromes: *ERCC3* with XP and TTD, *ERCC5* with XP and COFS, and *ERCC1* with COFS and XFEPS; all of them also involved in the XP/CS complex. Other genes linked to two distinct syndromes include *DNA2, CTC1, ATP6V0A2, ACD, DKC1, RTEL1, TERT,* and *TINF2*. Specifically, *DNA2* is associated with RTS and SCKL, *CTC1* with CRMCC and DKC, and *ATP6V0A2* with CL and WSS. The genes *ACD, DKC1, RTEL1, TERT,* and *TINF2* are all linked to both DKC and HHS, with the latter also considered a severe clinical form of DKC. Seven genes – *ALDH18A1, FBLN5, PYCR1, ACD, RTEL1, TERT,* and *TINF2* – are associated with two different subtypes within the same syndrome. Specifically, *ALDH18A1* is associated with ARCL3A and ADCL3, *FBLN5* with ARCL1A and ADCL2, *PYCR1* with ARCL2B and ARCL3B, *ACD* with DKCA6 and DKCB7, *RTEL1* with DKCA4 and DKCB5, *TERT* with DKCA2 and DKCB4, and *TINF2* with DKCA3 and DKCA5.

Among the 56 compiled syndromes, 18 have defined subtypes. The syndrome with the greatest number of subtypes is FA (22), followed by DKC (16), CL (14), AGS, TTD, and SCKL (each with 9), XP (8), MVA (6), BBS/CGL (5), RTS and COFS (4), CRMCC and EDSSPD (3), and DM, ATLD, CS, MAD, and RSDM (each with 2). Counting each syndrome without subtypes (38) and each gene-specific subtype of genetically heterogeneous syndromes as a separate entity results in a total of 160 distinct clinical entities. The syndrome with the greatest number of associated genes is FA (22), followed by DKC (15), SCKL (13), CL (11), XP, TTD and AGS (9), MVA (6), BBS/CGL and HHS (5), RTS and COFS (4), CRMCC and EDSSPD (3), and ATLD, CS, XFEPS, DM, APSs/AWS, MAD, and RSDM (each with 2). The remaining syndromes are associated with only a single gene. Details on the subtypes and gene associations are listed in Supplementary Table 1. Notably, the number of subtypes within a given syndrome does not necessarily reflect the number of genes associated with that syndrome, as some genes are linked to multiple subtypes within the same syndrome, whereas certain genes associated with a given syndrome could not be assigned to a specific subtype, resulting in the absence of subtype-level classification for these genes in the present catalogue.

Among all analyzed syndromes, SCKL is associated with the greatest number of clinical feature groups, covering 16 out of 18 defined categories; the only exceptions are the groups “neoplasia” and “immunology”. Among the SCKL subtypes, SCKL10 is associated with the largest number of groups, comprising 11 distinct clinical feature categories. Overall, the “head and neck” category is associated with the greatest number of syndromes and subtypes (i.e., distinct clinical entities), with a total of 150 connections. This is followed by: “skin, nails and hair” and “growth” with 130 connections, “neurologic” with 117, “skeletal” with 109, “genitourinary” with 74, “abdomen” with 72, “cardiovascular” with 58, “hematology” with 52, “muscle, soft tissues” with 50, “endocrine features” with 37, “chest” with 35, “respiratory” with 34, “neoplasia” with 28, “immunology” with 27, “prenatal manifestations” with 16, “voice” with 9, and “metabolic features” with 5.

### PPI network, functional enrichment and hub genes prioritization

**Figure 3:**
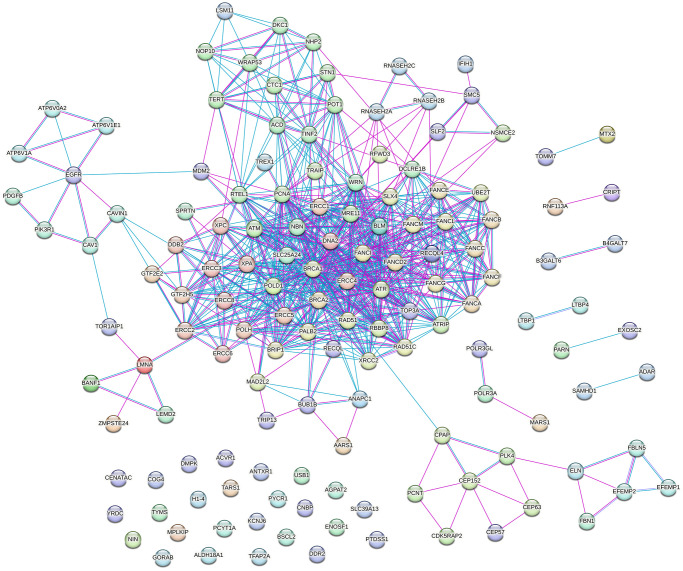
**Protein–protein interaction (PPI) network of proteins encoded by genes associated with progeroid syndromes.** The network was visualized using the STRING tool and includes only known interactions: blue edges represent interactions from curated databases, while pink edges indicate experimentally determined interactions. The network comprises 142 nodes and 720 edges, with an average node degree of 10.1 and an average local clustering coefficient of 0.618. The PPI enrichment *p*-value is <1.0 × 10^−^¹^6^.

To investigate the functional and physical relationships (i.e., interactions) among proteins encoded by genes associated with the compiled syndromes, we constructed a PPI network using the STRING database. Known interactions among 142 proteins linked to these syndromes are visualized in Figure 3.

The resulting network consists of 142 nodes and 720 edges, with an average node degree of 10.1 and an average local clustering coefficient of 0.618. The calculated PPI enrichment *p*-value is <1.0 × 10^−^¹^6^, indicating that the observed number of interactions (720) is significantly higher than the expected number (108) for a network of this size. This suggests that the proteins in the network are at least partially biologically connected and are likely to participate in related biological pathways or cellular processes.

**Figure 4:**
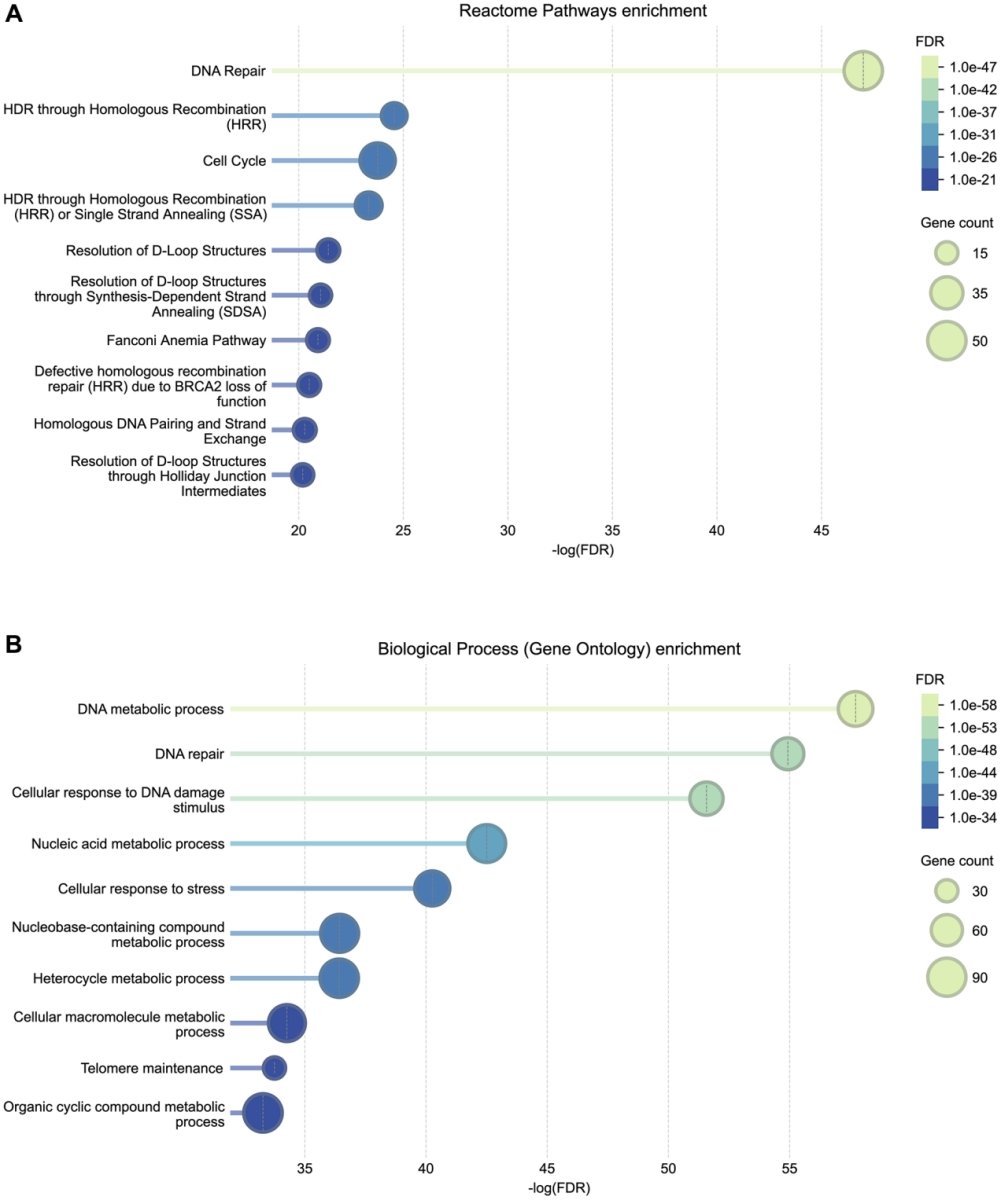
Reactome pathway (**A**) and Gene Ontology (GO) Biological Process (**B**) functional enrichment of genes associated with progeroid syndromes. Bubble plots show the top ten enriched biological pathways and processes identified from STRING’s functional enrichment outputs. The x-axis represents the –log(FDR) significance of enrichment. Bubble size corresponds to the number of genes annotated to each pathway/process, and bubble color reflects the adjusted FDR value. Complete enrichment results are provided in Supplementary Tables 3 (GO) and 4 (Reactome).

STRING’s functional enrichment analysis identified a total of 354 significantly enriched (FDR <0.05) Gene Ontology (GO) Biological Process terms (Supplementary Table 3) and 91 significantly enriched Reactome pathways (Supplementary Table 4) represented within the constructed PPI network. Examination of the top ten most significantly enriched Reactome pathways revealed a strong and specific over-representation of processes related to DNA repair and genome maintenance (Figure 4A). “DNA Repair” was the most significantly enriched pathway (FDR = 1.01 × 10^−47^), involving 50 genes from the network. Highly enriched pathways included multiple homologous recombination-associated processes, pathways describing the resolution of D-loop structures, as well as the “Fanconi Anemia Pathway”. A broader enrichment of “Cell Cycle” processes was also observed. Gene Ontology (GO) Biological Process enrichment (Figure 4B) further supported the central role of genome maintenance pathways in the analyzed gene set. The most significantly enriched terms included “DNA metabolic process” (FDR = 1.9 × 10^−58^) and “DNA repair,” both involving large subsets of the network. Additional highly enriched processes comprised “Cellular response to DNA damage stimulus,” “Nucleic acid metabolic process,” and broader stress response and DNA metabolism-related pathways. Additionally, “Telomere maintenance” was among the top ten significantly over-represented terms.

Beyond the top-ranked pathways and processes predominantly related to DNA repair and telomere maintenance, additional significantly enriched terms were identified in the complete enrichment results (Supplementary Tables 3 and 4). These include pathways and processes related to gene expression and transcriptional regulation, multiple cell cycle-related processes, including regulation of cell cycle and checkpoint control, DNA replication, as well as various cellular stress responses and cellular senescence. Enrichments were also observed for terms linked to extracellular matrix organization and elastic fiber-associated components, processes involved in protein metabolism and post-translational modifications, organelle biogenesis and maintenance, as well as nuclear envelope organization and reformation. Furthermore, signaling and regulatory pathways, including insulin receptor signaling, along with RNA metabolic and epigenetic processes and immune-related pathways, were also among the significantly enriched terms. While a broad range of biological processes and pathways was detected, the most statistically significant enrichments consistently corresponded to processes involved in maintaining DNA integrity and genome stability.

**Figure 5:**
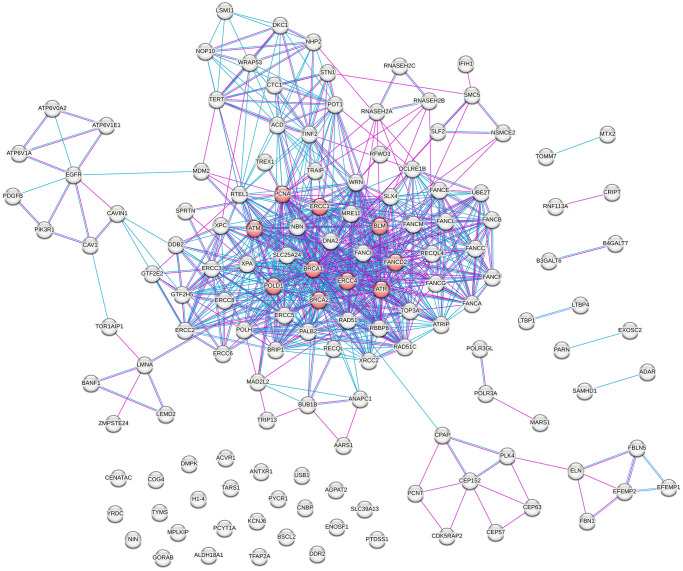
**Protein–protein interaction (PPI) network of proteins encoded by genes associated with progeroid syndromes, highlighting prioritized hub proteins.** Hubs, defined as the top ten nodes ranked by node degree, are shown in red, representing proteins with a high number of interaction partners and central positions within the network topology.

To further characterize the constructed PPI network and identify highly connected nodes, we prioritized hub genes/proteins based on node degree. The ten proteins with the highest number of interaction partners were BRCA1 (degree = 56), ERCC4 (39), ERCC1 (38), ATR (35), ATM (34), FANCD2 (34), PCNA (34), POLD1 (34), BRCA2 (33), and BLM (32) (Table 1). As shown in Figure 5, these proteins are predominantly located in the central, densely connected region of the network, where they engage in numerous interactions with other proteins. Cross-referencing the corresponding hub genes with Open Genes, a curated database of human genes associated with aging and longevity, revealed that nine of the ten prioritized hub genes are included within the database for their evidence-based links to aging-related processes and phenotypes beyond rare progeroid syndromes. *FANCD2*, although highly connected within the network, was not present in Open Genes at the time of analysis (Table 1). Detailed information on the evidence and selection criteria used for gene inclusion and curation as aging-related for each gene marked as “yes” in Table 1 is available through the Open Genes database (https://open-genes.com).

**Table 1:** Prioritized hub genes in the progeroid-associated protein–protein interaction (PPI) network.

**Gene**	**Node degree**	**Included in Open Genes database**
*BRCA1*	56	yes
*ERCC4*	39	yes
*ERCC1*	38	yes
*ATR*	35	yes
*ATM*	34	yes
*FANCD2*	34	no
*PCNA*	34	yes
*POLD1*	34	yes
*BRCA2*	33	yes
*BLM*	32	yes

Hub genes were identified based on node degree, defined as the number of direct interaction partners within the PPI network. The table lists the ten genes with the highest degree values, along with their corresponding node degrees and inclusion status in Open Genes, a curated database of human genes associated with aging and longevity. Detailed evidence supporting the links to aging-related biology for each gene marked as “yes” is available through the Open Genes database (https://open-genes.com).

### Case study: *LMNA* gene and associated diseases

As a case study, we examined the phenotypic diversity arising from single-gene variants in the *LMNA* gene. Supplementary Table 5 provides a list of 12 human disorders caused by variants in the *LMNA* gene, extracted from five key publications [23–27] and the OMIM database. The table also includes the associated clinical feature groups, totaling 15 distinct categories, defined according to the organ systems or body parts affected, as listed in the OMIM database. Among the 12 disorders included in our dataset, four are traditionally classified as progeroid syndromes, whereas the remaining eight fall within other phenotypic categories – including lipodystrophies, neuropathies, cardiomyopathies, and muscular dystrophies.

**Figure 6:**
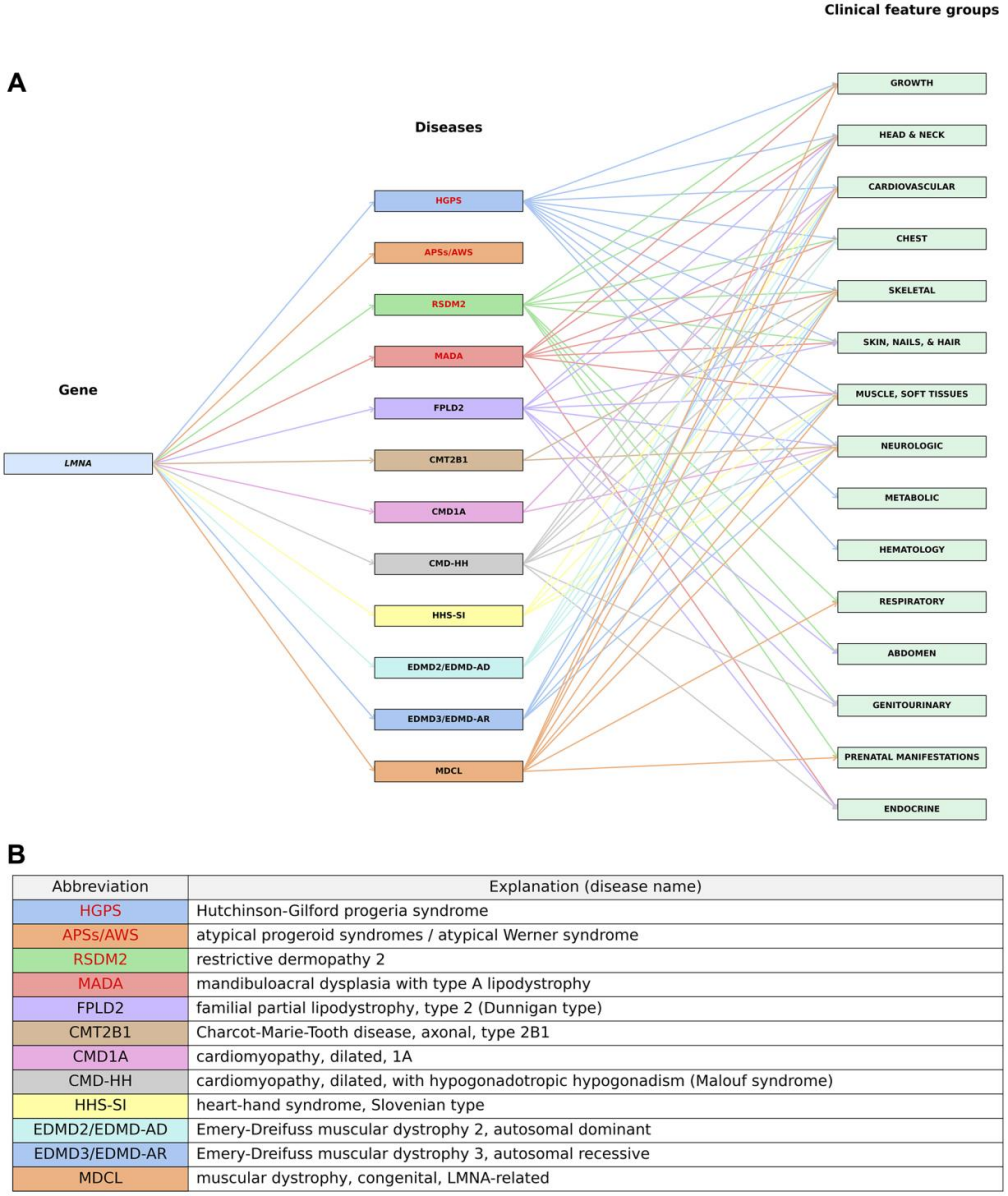
**A graphical visualization of associations between the *LMNA* gene, associated diseases and clinical feature groups.** (**A**) The *LMNA* gene (left) is connected to its associated diseases (middle), which are further linked to their corresponding clinical feature groups (right). Progeroid syndromes are highlighted in red. (**B**) Explanations of disease abbreviations (full disease names). Details are provided in Supplementary Table 5.

The compiled data were visualized in the form of a genome–phenome association network (Figure 6), depicting the associations between the *LMNA* gene, the 12 associated diseases, and their respective clinical feature groups (Figure 6A). Explanations of disease abbreviations (disease names) are provided in the legend accompanying the network (Figure 6B). Figure 6 aims to illustrate the involvement of the *LMNA* gene in a variety of disorders, extending beyond progeroid syndromes, yet often exhibiting overlapping phenotypes. Together, this example demonstrates how a single gene can underpin a wide spectrum of disorders with partially overlapping features, reflecting complex genotype–phenotype relationships and underscoring broader patterns of pleiotropy across progeroid syndrome genes.

## DISCUSSION

By integrating data from existing literature and the OMIM database, we developed a manually curated resource on progeroid and related premature aging syndromes. The resulting PS catalogue provides, to our knowledge, the largest published collection of such disorders to date, encompassing 144 genes, 56 syndromes, and a total of 160 distinct clinical entities. In addition to genes and syndromes, the dataset also includes major clinical feature groups, defined according to affected organ systems or body regions, associated with each of the included clinical entities. Visualization of the compiled data through a genome–phenome association network provides an overview of the genetic heterogeneity underlying these conditions, which is reflected in phenotypically diverse syndromes with partially overlapping clinical profiles. Complementary analyses using a PPI network and functional enrichment further reveal that the associated proteins form a highly interconnected network with pronounced enrichment of genome maintenance pathways. Within this network, prioritization of highly connected hub genes and their cross-referencing with the Open Genes database revealed that nine of the ten are currently curated within the database with evidence-based links to aging-related biology beyond rare progeroid syndromes, adding an additional layer of contextualization for the PPI network. Together, these integrative analyses provide a framework to unify previously fragmented knowledge, highlight opportunities to refine classification, support diagnostics, and advance our knowledge of premature aging syndromes and their broader implications for physiological human aging. In the following sections, we first outline the main challenges encountered during the development of this resource, followed by key insights derived from the integrated data. Finally, we discuss the limitations of the current catalogue version and propose future directions for its expansion and broader application.

### Unifying scattered data: challenges in classification and terminology

Some of the main challenges encountered during the assembly of this resource stemmed from variations in terminology and differences in classifications of genes and diseases across scientific sources on progeroid syndromes, reflecting historical differences in nomenclature and clinical classification frameworks. For example, the same gene is frequently reported under different symbols – including previous, alias, or unofficial – in various publications and/or in the OMIM database, complicating efforts to retrieve and compare genotype–phenotype data in an already broad and fragmented field. To address this, we standardized all gene names and symbols according to the approved HGNC nomenclature, while also recording previous and alias symbols used in the cited literature. This approach not only ensures a clearer overview of included genes but also facilitates downstream bioinformatic analyses that typically require a consistent and standardized input gene list.

A further challenge concerned the classification of syndromes into subtypes. Across the literature, different frameworks are used: while some studies divide individual syndromes into subtypes, each associated with a specific causal gene, others describe them as single syndromes with multiple genetic drivers, still others adopt hybrid approaches. Since the OMIM database provides a curated categorization of syndrome subtypes (i.e., gene-specific phenotypes within genetically heterogeneous disorders), we used it as the primary framework for building our catalogue. However, this introduced an additional challenge: some gene–syndrome associations reported in the literature were not listed in OMIM at the time of curation, preventing their assignment to corresponding subtypes and to the associated clinical profiles extracted from OMIM.

Another challenge in classifying progeroid syndromes arises from the substantial clinical overlap among certain conditions, which further contributes to differences in classification between sources. As an example, geroderma osteodysplastica (GO), cutis laxa (CL), and wrinkly skin syndrome (WSS) have been variably classified in the literature due to strong phenotypic similarities: GO has been categorized as a progeroid form of CL, GO and WSS have been proposed to represent the same disorder, while WSS and some cases of CL have been described as a continuum of a single disorder with varying severity [15, 56, 59, 97–99]. Such cases were documented in the “Notes” section of the developed resource. Additionally, phenotypic overlaps between progeroid syndromes and other hereditary disorders not traditionally classified as progeroid (discussed below) further complicate classification boundaries. Importantly, as new genetic and clinical evidence emerges, further refinements and changes in gene–phenotype associations and syndrome and subtype classifications can be expected.

Through the consolidation of terminology and classifications, we aimed to establish a cohesive framework to support future research in syndrome classification, comparative analyses, and improved understanding of genotype–phenotype relationships, with potential relevance for diagnostics and therapeutic development. Importantly, our catalogue includes both well-established and newly discovered or proposed progeroid syndromes. In some cases, the gene–phenotype associations in OMIM are labeled as “provisional”, or certain syndromes included in our catalogue were only proposed as progeroid in the literature based on premature or accelerated aging phenotypes. These cases were likewise annotated in the catalogue. While some of these associations and classifications may be subject to further refinement or reclassifications as additional evidence becomes available, we believe that this broader approach enables a more comprehensive view of the current state of the field and may ultimately provide deeper insights into potential mechanisms of (premature) aging.

### New insights into genetic and phenotypic heterogeneity of progeroid syndromes

Progeroid syndromes are widely recognized as a genetically and phenotypically heterogeneous group of disorders [12, 15, 17, 91], a characterization that is clearly supported by our dataset and the genome–phenome association network visualization and analysis.

#### Genetic heterogeneity and pleiotropy

Our genome–phenome association network analysis revealed that genetic heterogeneity within progeroid syndromes manifests at different levels: on the one hand, pathogenic variants in multiple distinct genes can lead to different subtypes of the same syndrome; on the other hand, the same gene, depending on the type of pathogenic variant, can contribute to the pathogenesis of multiple different syndromes or their subtypes.

We found that 18 out of the 56 compiled syndromes have defined subtypes. The presence of subtypes likely reflects the fact that these syndromes were initially described based primarily on clinical features, while later molecular studies uncovered multiple causal genes in many cases, with pathogenic variants in any one of them producing a similar or indistinguishable phenotype (locus heterogeneity of monogenic disorders) [100]. These genes typically act within the same or overlapping molecular pathways, indicating a functional relationship in disease development [10, 100]. However, since clinical presentation within a single syndrome can vary significantly depending on the specific genetic variant, Koschitzki et al. (2023) have already pointed out the need for updated classification in the case of trichothiodystrophy (TTD) [16]; similarly, we observed such variability in several other syndromes and their subtypes (discussed below).

The network analysis also revealed that 19 of the 144 genes in our dataset are associated with more than one syndrome or syndrome subtype. For example, different pathogenic variants in the *LMNA* gene are associated with multiple progeroid phenotypes that differ from classical Hutchinson-Gilford progeria syndrome (HGPS), including restrictive dermopathy type 2 (RSDM2), mandibuloacral dysplasia with type A lipodystrophy (MADA), and atypical progeroid syndromes (APSs). It is known that regardless of whether a *LMNA* variant leads to the production of progerin or not, it disrupts the nuclear lamina and causes a spectrum of progeroid phenotypes in varying severity; moreover, studies have shown that different types of *LMNA* variants can result in milder or more aggressive forms of HGPS, depending on the amount of progerin produced [14, 27, 68]. Similarly, in patients with progeroid syndromes, nearly 30 different pathogenic variants in the *ZMPSTE24* gene have been described, leading to milder or more severe phenotypes of RSDM1, MADB, or APSs/AWS [14]. Several studies also address the involvement of different pathogenic variants in various pleiotropic genes involved in the nucleotide excision repair (NER) pathway – including *ERCC1, ERCC2* (*XPD*), *ERCC3* (*XPB*), *ERCC4* (*XPF/FANCQ*), *ERCC5* (*XPG*), and *ERCC6* (*CSB*) – across multiple overlapping disorders, such as xeroderma pigmentosum (XP), XFE progeroid syndrome (XFEPS), trichothiodystrophy (TTD), Fanconi anemia (FA), Cockayne syndrome (CS), cerebro-oculo-facio-skeletal syndrome (COFS), DeSanctis-Cacchione syndrome (DSC), and rare combinations such as XP/CS and XP/TTD complexes [6, 13, 37, 42, 46, 60]. Furthermore, pathogenic variants in *CTC1* are associated with two telomeropathies: cerebroretinal microangiopathy with calcifications and cysts 1 (CRMCC1) and dyskeratosis congenita (DKC), while each of the genes *ACD, DKC1, RTEL1, TERT,* and *TINF2* is involved in multiple subtypes of DKC as well as its severe clinical form, Hoyeraal-Hreidarsson syndrome (HHS). Genes such as *ALDH18A1, PYCR1,* and *FBLN5* can cause different subtypes of cutis laxa (CL), while *ATP6V0A2* is associated with either CL or wrinkly skin syndrome (WSS). The gene *DNA2* is linked to both Seckel syndrome type 8 (SCKL8) and Rothmund-Thomson syndrome type 4 (RTS4).

In addition to their involvement in multiple progeroid syndromes, the genes implicated in these syndromes also contribute to the pathogenesis of other diseases, as demonstrated in the case of the *LMNA* gene. To date, nearly 500 different pathogenic variants in *LMNA* have been described, with a continuously updated list available in the UMD-*LMNA* mutations database (http://www.umd.be/LMNA/). These variants lead to a range of human disorders, collectively termed primary laminopathies, which include not only premature aging syndromes but also lipodystrophies, neuropathies, cardiomyopathies, and muscular dystrophies with partially overlapping phenotypes, which can sometimes make them difficult to distinguish [14, 23–27]. Numerous case reports and familial studies have documented additional diverse, yet unclassified, phenotypes associated with *LMNA* variants, many of which share features with recognized laminopathies. Importantly, although the disorders listed in our collection and *LMNA*-network are traditionally regarded as distinct entities, there is often significant phenotypic overlap, with some individuals and families exhibiting features of multiple laminopathies. Furthermore, identical *LMNA* variants can result in diverse phenotypes across unrelated individuals, underscoring the complexity of genotype–phenotype relationships. Despite extensive research, the mechanisms by which *LMNA* mutations lead to such diverse disease manifestations remain unclear, though several hypotheses have been proposed [23–25, 27]. Similar patterns of a single gene driving multiple diseases with overlapping phenotypes could be illustrated for many other genes listed in our catalogue.

#### Phenotypic heterogeneity and overlap

Progeroid syndromes also exhibit significant phenotypic heterogeneity, affecting various organ systems and body parts, as illustrated by our genome–phenome network. Although all progeroid syndromes display certain clinical features of premature aging, the network reveals that the specific organ systems affected differ between syndromes – and even between subtypes of the same syndrome. While this study focused only on major clinical feature groups defined by affected organ systems or body regions, such categorization already highlights the diverse range of clinical manifestations observed in progeroid syndromes and underscores the variability between syndromes and subtypes, while keeping the presentation concise.

It is important to emphasize, however, that clinical heterogeneity extends beyond organ-specific involvement as presented in our visualization. Even within the defined clinical feature groups, specific clinical signs and symptoms vary by syndrome or subtype, and the same sign or symptom may manifest with varying severity across different conditions. As a result, some disorders present with milder forms, while others progress aggressively [14]. Furthermore, even within a single syndrome, individual patients can display different clinical profiles: some features may be nearly universal, while others are rare [91]. Additionally, even unrelated individuals carrying the same pathogenic variant may not exhibit identical clinical presentations; beyond the *LMNA* gene, this phenomenon has been for example well documented in Cockayne syndrome, where patients harboring identical variants in *ERCC8* (*CSA*) [101] or *ERCC6* (*CSB*) [102] can present with markedly diverse clinical phenotypes. This variability points to a complex genotype–phenotype relationship and suggests that clinical expression is not determined solely by the genetic variant but is also influenced by other genetic, environmental, or epigenetic factors.

On the other hand, as already noted, many progeroid syndromes also exhibit overlapping phenotypes, which complicates both the classification and clinical diagnosis [12, 14, 16, 19, 91]. For example, wrinkly skin or cutis laxa is a common feature across several syndromal disorders, including autosomal dominant and recessive cutis laxa (ADCL, ARCL), wrinkly skin syndrome (WSS), and geroderma osteodysplastica (GO), leading to classification and diagnostic challenges due to broad clinical overlap [56, 80]. Additionally, variants in the same gene suggest that WSS and ARCL2A may represent variable manifestations of a shared genetic defect, although key differentiating features remain critical [59, 97]. Furthermore, phenotypic overlaps between syndromes traditionally classified as progeroid and other conditions – such as Seckel syndrome (SCKL) sharing phenotypic features with other disorders characterized by microcephaly and/or primordial dwarfism, or dyskeratosis congenita (DKC) with other telomere-related disorders or “inherited poikilodermas” – can lead to discrepancies in gene–syndrome associations, complicating their classification and diagnosis [77, 89, 90]. The presence of shared clinical features across multiple disorders makes precise syndrome delineation challenging and, as noted above, some of the reported associations and classifications may remain subject to ongoing discussion and refinement.

### Functional insights from the PPI network and enrichment analysis

The clinical diversity of progeroid syndromes reflects the complex relationship between genetic variants and their diverse effects on individual organ systems. The type and severity of symptoms can vary depending on the affected gene and the altered molecular pathway [11], as well as on specific variants within these genes, as discussed above. On the other hand, the overlap of clinical features across several progeroid syndromes points to shared or related molecular mechanisms that contribute to the pathogenesis of these disorders [15, 103]. All progeroid syndromes share a phenotype of premature aging, and it is known that, in addition to clinical signs, they also share molecular and cellular characteristics with physiological aging [4–6, 11–13, 17, 19, 61]. To examine these shared mechanisms within our curated gene set, we conducted PPI network and functional enrichment analyses.

The constructed PPI network revealed a strong interconnectedness among involved proteins, with a PPI enrichment *p*-value <1.0 × 10^−^¹^6^, indicating that these proteins likely participate in shared biological pathways or cellular processes. Consistent with the published literature, the functional enrichment analysis performed across all GO Biological Process terms and Reactome pathways within STRING showed that the compiled gene set is predominantly enriched in DNA repair and associated genome maintenance pathways. Indeed, it is well established that many progeroid syndromes arise from defects in diverse mechanisms safeguarding genome integrity, including homologous recombination, interstrand crosslink repair (Fanconi anemia pathway), nucleotide excision repair, DNA damage response signaling, and telomere maintenance [3, 4, 6, 9, 11, 13–18], and the same processes were strongly enriched in our analysis. The strong overrepresentation of DNA repair pathways in our dataset is consistent with the view that genomic instability plays a key mechanistic role in many progeroid syndromes, paralleling its established role in physiological aging. This interpretation aligns with the classification of genomic instability as a primary hallmark of aging and with the well-established decline in DNA repair efficiency over the lifespan, which contributes to the accumulation of genomic lesions and subsequent age-related physiological decline [1, 8].

In addition to DNA repair pathways, significant enrichment was also observed for processes related to telomere maintenance, consistent with telomere attrition as another primary hallmark of aging [1, 8]. Several cell cycle- and gene expression-related processes were also enriched, in line with their dysregulation during physiological aging. Disruptions in cell cycle control and cellular stress responses are known to contribute to the induction of cellular senescence, an antagonistic hallmark of aging [1, 8], while altered gene expression and transcriptional regulation broadly impact cellular homeostasis. Enrichment of terms associated with nuclear envelope organization further aligns with the recognized role of nuclear architecture defects in aging and highlights nuclear envelope-related progeroid syndromes as a major group within these disorders [3, 4, 6, 9, 11, 13–18]. Additional enriched processes corresponded to other established hallmarks of aging, including extracellular matrix organization associated with altered intercellular communication, immune-related pathways reflecting immunosenescence and chronic inflammation, and the insulin receptor-related signaling pathway linked to deregulated nutrient sensing [1, 8]. Several pathways involved in protein metabolism, post-translational modifications, and organelle biogenesis and maintenance were also significantly represented, consistent with age-associated loss of proteostasis and organelle dysfunction [1, 8]. Although a broad spectrum of biological functions was represented among the enriched terms, DNA repair and genome maintenance pathways consistently exhibited the strongest statistical support in the present dataset, underscoring their particular relevance within the analyzed gene set and aligning with the view that compromised genome stability constitutes a major shared feature across many progeroid syndromes and an important link between these syndromes and physiological aging.

Prioritization and validation of highly connected genes within the PPI network provided additional context for the broader aging relevance of progeroid-associated genes. Ranking genes by node degree of their corresponding proteins within the network identified a set of highly connected hub genes, including *BRCA1, ERCC4, ERCC1, ATR, ATM, FANCD2, PCNA, POLD1, BRCA2,* and *BLM*. Cross-referencing these hub genes with Open Genes, a curated database of human genes associated with aging and longevity, showed that nine of the ten most highly connected genes are included within the database for their links to aging-related processes and phenotypes beyond rare progeroid syndromes. In Open Genes, these associations are supported by diverse lines of evidence, as defined by the database’s selection criteria, including age-related changes in gene expression, methylation, or protein activity in humans and model organisms, experimental modulation of gene activity affecting lifespan or age-related processes, associations of genetic variants or gene expression levels with longevity, association of genes with accelerated aging in humans, and regulation of genes linked to aging [104].

Notably, all nine hub genes included in Open Genes are associated with the hallmark of aging “nuclear DNA instability” within the database, consistent with the strong enrichment of genome maintenance-related pathways observed in our PPI-based analysis. Several, but not all, of these genes are additionally linked to other hallmarks of aging, including telomere attrition, epigenetic and transcriptional alterations, proteostatic decline, TOR pathway dysregulation, and accumulation of senescent cells [104]. While *FANCD2* was the only hub gene not included in Open Genes at the time of analysis, its relevance to aging biology is supported by its established role in the Fanconi anemia pathway and interstrand crosslink DNA repair, processes tightly connected to aging-related genome instability when disrupted [105]. It is important to note that validation against Open Genes was limited to the subset of highly connected hub genes, and systematic comparison of the full curated gene set with aging and longevity databases therefore represents an important direction for future work. Nonetheless, this prioritization highlights a set of highly connected proteins at the center of the progeroid-associated PPI network with established links to aging-related biology, further supporting the broader relevance of the constructed network.

### Limitations and future perspectives

Given the expansive scope of premature aging disorders and the continual discovery of new genes and syndromes, we acknowledge that our dataset is inherently incomplete. This dynamic nature of the field is reflected in recent discoveries emerging after the literature cutoff used in this study, as well as updates to curated databases such as OMIM following the data curation period, for example the identification of an additional xeroderma pigmentosum complementation group (XP-J) [106], underscoring the need for periodic updates of curated resources. At the same time, it is important to note that despite recent advancements in next-generation sequencing (NGS) technologies, which have uncovered the causative pathogenic variants of various atypical premature aging syndromes, the genetic cause in some cases remains unclear [14]. Two examples include Hallermann-Streiff syndrome [15] and Mulvihill-Smith syndrome [5], which were not included in our dataset due to the unknown genetic cause. Additionally, as syndrome definitions, classifications, and associated terminology continue to evolve as new molecular and clinical evidence emerges, some gene–syndrome associations included in the catalogue may be subject to future discussions, refinement or reclassification. Continued refinement and expansion of this dataset are essential for future research, aiming to provide a more comprehensive understanding of the genetic and molecular landscape of premature aging syndromes.

In addition to expanding the gene set, it would be beneficial to enrich the catalogue with more detailed phenotypic descriptions for each syndrome and subtype, including detailed characterization of specific progeroid or premature aging features. Given that phenotypic expression can vary depending on the specific gene and variant involved, not all genes or variants associated with a given syndrome may contribute equally to progeroid or premature aging features. Including such information could facilitate deeper exploration of complex genotype–phenotype relationships, improve understanding of the effects of individual gene variants, help clarify and/or verify their contribution to progeroid or premature aging features, enable more precise comparisons between syndromes, and support more accurate classification, diagnostics, and ultimately treatment of individual progeroid conditions.

In this study, we primarily focused on the DNA level – specifically, causative genes responsible for progeroid and related premature aging syndromes. While these disorders are predominantly monogenic in origin, the downstream molecular consequences extend across multiple biological layers. Future efforts could expand the dataset by incorporating information on genetic modifiers, as well as additional omics layers – including epigenomic, transcriptomic, proteomic, and metabolomic data – that influence phenotypic outcomes beyond the primary disease-causing variants. Integrating these multi-omics dimensions could provide a more comprehensive view of disease mechanisms, reveal regulatory factors that modulate disease severity or progression, and help identify novel biomarkers and therapeutic targets.

Additionally, the compiled gene set could be explored through more fine-grained functional analyses. Beyond the initial analyses presented here, a deeper and more specific investigation of the associated biological pathways, including more detailed comparative analyses with physiological aging, could further clarify mechanisms shared between these syndromes and “natural” aging and reveal points of convergence that could serve as potential therapeutic targets. Further examination of the constructed PPI network, as well as of the genomic distribution of the associated genes, may also help highlight critical molecular and genomic “hotspots” and identify novel candidate genes underlying these disorders. Systematic validation of the full gene set using independent datasets, including expression-based resources, could additionally help prioritize key genes and strengthen the biological relevance of the catalogue. Moreover, future re-analyses incorporating newly identified genes, along with increasingly complete and curated data on biological processes, pathways, and molecular interactions from publicly available databases such as STRING, Reactome, and Gene Ontology, may reveal additional insights that are not captured in the present study.

Finally, we believe that continued identification and analysis of causal genes and their associated molecular pathways may further advance our understanding of fundamental aging processes and, in doing so, open new avenues for the development of biomarkers and therapeutic strategies – potentially benefiting not only individuals with progeroid syndromes but also those affected by other age-related diseases.

## CONCLUSIONS

In this work, we present a curated catalogue of 144 genes associated with 56 premature aging-related syndromes and a total of 160 distinct clinical entities, representing, to our knowledge, the largest compilation of these disorders to date. By consolidating previously scattered data on genetic factors, applying categorization, and standardizing gene terminology, this resource addresses challenges arising from fragmented and often inconsistent data in the field and provides a reference for future research. Visualizing the dataset as a genome–phenome association network offers new insights into the multi-level genetic and phenotypic heterogeneity of these syndromes, and it may also serve as a transferable model for depicting and analyzing genome–phenome relationships in other complex disorders. The constructed PPI network revealed strong interconnectivity among the involved proteins, and functional enrichment highlighted DNA repair and associated genome maintenance pathways as the most strongly enriched, consistent with the established role of accumulated DNA damage in both progeroid syndromes and physiological aging. In addition, prioritization of highly connected hub genes within the PPI network, together with their cross-referencing with the Open Genes database, highlighted central network components with previously reported links to aging-related biology beyond rare progeroid syndromes. Ongoing expansion of this resource, together with additional analyses of the compiled data, may uncover further insights into the genetic architecture and mechanisms of premature aging, with potential implications for a better understanding of physiological aging. Continued investigation of progeroid syndromes holds potential for informing strategies to address both progeroid conditions and, potentially, other aging-related diseases.

## MATERIALS AND METHODS

To establish a unified framework for studying genetic disorders exhibiting features of premature aging, we manually compiled, curated, and subsequently visualized existing data on progeroid and related syndromes and their associated genes. We then analyzed the compiled gene set through PPI network construction, functional enrichment analysis, and hub gene prioritization, and illustrated phenotypic diversity arising from single-gene variants in the compiled genes by examining the disease spectrum associated with the *LMNA* gene.

### Progeroid syndrome catalogue (PS catalogue) compilation

To construct the PS catalogue, genes associated with the phenotype of progeroid syndromes were compiled from scientific publications accessible through the NCBI PubMed database [107] and from the Online Mendelian Inheritance in Man (OMIM) database [108]. Initial queries for identifying relevant literature in PubMed included the terms “progeria,” “progeroid,” and “premature aging,” with a restriction to literature published between January 2000 and December 2024. Inclusion criteria required a reported association between a gene and a syndrome classified as progeroid or a disorder reported to exhibit progeroid, premature aging or accelerated aging features. Studies describing phenotypes without linking them to specific genes, as well as progeroid syndromes with unknown genetic causes or those resulting from chromosomal imbalances, were excluded, as it was not possible to establish a clear gene–phenotype association. Both original research papers and review articles were included, and further studies were identified through reference lists within the included publications.

Identified genes and their corresponding syndromes were recorded in a table (i.e., catalogue). Each recorded gene was verified manually in OMIM (accessed between December 2024 and February 2025) and assigned to the corresponding syndrome subtype (i.e., a genetically defined phenotype – e.g., form, type, or complementation group – within a genetically heterogeneous disorder, as represented in OMIM phenotypic series) according to that database. If a gene–syndrome association could not be assigned to a specific subtype based on the available OMIM annotations at the time of curation, such association was nevertheless included if documented in at least two other supporting literature sources, and the corresponding subtype designation was recorded as “NA”. Where missing, additional genes and subtypes for a given syndrome previously recorded in the catalogue were obtained from OMIM, and associations between newly added genes and respective syndromes were further verified through PubMed queries using specific search terms for each gene–syndrome pair. To further expand the dataset, we conducted an advanced search in OMIM using the query “progeria” OR “progeroid,” combined with the MIM prefix “# phenotype description, molecular basis known”. This search yielded additional genes and syndromes, whose classification as progeroid or association with premature aging-related features was again confirmed through PubMed searches. References supporting an association between a specific gene and a progeroid syndrome or a disorder exhibiting progeroid, premature aging or accelerated aging features were recorded in a dedicated column of the established catalogue; at least two supporting references per gene–syndrome association were required and documented.

Gene symbols and names in the catalogue were standardized according to the HUGO Gene Nomenclature Committee (HGNC) database [109]. Alongside the approved symbols and names, previous and/or alias symbols used in the cited literature were also included for reference. Finally, for each syndrome or its subtype, associated clinical features were extracted from the OMIM database. The entries were limited to the major clinical feature groups – defined according to the organ systems or body parts affected – as listed in OMIM (“clinical synopses”), ensuring a concise yet representative overview of the clinical features of each condition. In accordance with distinct clinical characterization of each genetically defined phenotype in OMIM, clinical entities were defined as individual syndromes without subtypes and gene-specific subtypes of genetically heterogeneous syndromes, with each counted as a separate entity.

Additionally, to facilitate subsequent analyses, a separate gene list of the compiled genes was prepared, containing approved gene symbols, names, genomic locations, cytogenetic bands, and corresponding Ensembl, NCBI, and HGNC identifiers (IDs). Gene-related data were obtained using the Ensembl BioMart data-mining tool (Ensembl release 113; [110, 111]), based on the GRCh38.p14 genome assembly.

### Data visualization and analysis: the genome–phenome association network

To visually represent the relationships between the genes, compiled syndromes (or their subtypes), and clinical features (i.e., clinical feature groups), Python v3.9.6 was used along with the libraries Pandas v2.2.3 [112], NetworkX v3.2.1 [113], Seaborn v0.13.2 [114], and Matplotlib v3.9.4 [115]. Each gene from the compiled catalogue was linked to its corresponding syndrome or syndrome subtype, which was in turn linked to its associated clinical feature groups. To facilitate visual distinction between different syndromes – some of them represented by a set of distinct subtypes – we applied color mapping by syndrome. Based on the resulting visualization, we then conducted a network analysis to determine (i) how many and which genes are associated with more than one syndrome or subtype; (ii) which syndrome includes the greatest number of subtypes; (iii) which syndrome is linked to the greatest number of distinct genes; (iv) which syndrome and subtype is associated with the greatest number of clinical feature groups; and (v) which clinical feature group is shared by the greatest number of syndromes and subtypes.

### PPI network construction and analysis

To functionally characterize the compiled gene list, we first investigated the functional and physical relationships (i.e., interactions) among proteins encoded by genes associated with the compiled syndromes. A PPI network was constructed using the STRING v12.0 tool [116]. A total of 142 HGNC-approved symbols corresponding to protein-coding genes were uploaded, while the two non-coding RNA (ncRNA) genes from the original set were excluded. *Homo sapiens* was selected as the target organism. Active interaction sources were restricted to known interactions, including experimentally validated and curated database interactions; predicted interactions (gene neighborhood, gene fusion, and gene co-occurrence), as well as interactions inferred from text mining or co-expression, were disabled. The minimum required interaction score was set to 0.4 (medium confidence).

STRING’s built-in functional enrichment analysis outputs and visualizations were then examined to identify significantly enriched biological processes and pathways represented within the constructed network, focusing specifically on Gene Ontology (GO) Biological Process enrichment and Reactome pathway over-representation. Enriched terms were ranked by false discovery rate (FDR).

Furthermore, key hub genes were identified based on network centrality within the PPI network. Specifically, node degree, defined as the number of direct interaction partners for each protein, was used as a measure of degree centrality to identify highly connected hub nodes, as commonly applied in biological network analyses [117–120]. Node degree values were obtained directly from the STRING database, and proteins were ranked in descending order according to their degree. The ten genes encoding the proteins with the highest degree values were designated as putative hub genes. To assess their relevance to aging-related processes and phenotypes beyond progeroid syndromes, these genes were subsequently cross-referenced with Open Genes v2.2.0 (build 16585443221.108), a manually curated database of human genes associated with aging and longevity, which includes and curates aging-related genes based on predefined selection criteria as specified by the database [104].

### Case example: *LMNA* gene and associated diseases

To explore the phenotypic diversity arising from single-gene variants in the compiled genes, we constructed a table and network of *LMNA*-associated diseases and their clinical manifestations. Diseases associated with variants in the *LMNA* gene were extracted from a combination of key PubMed publications and the OMIM database. Associated clinical manifestations, i.e., clinical feature groups, defined according to the organ systems or body parts affected, were obtained from OMIM. The relationships between the *LMNA* gene, its associated diseases, and corresponding clinical feature groups were visualized using Python 3.9.6 as described above.

## Supplementary Materials

Supplementary Table 1

Supplementary Table 2

Supplementary Table 3

Supplementary Table 4

Supplementary Table 5
