# Supplementary material for: A manually curated gene–phenotype catalogue for progeroid syndromes and premature aging
Source: Aging (Albany NY). 2026 Mar 30;18(1):234–60. doi: 10.18632/aging.206366 (PMC13285954; doi:10.18632/aging.206366)
Supplement: Supplementary Table 1 [file aging-18-1-206366-s001.docx]

**Supplementary Table 1. Progeroid syndrome (PS) catalogue.**

| **Gene(s)** | **Syndrome** | **Subtype(s)** | **Clinical feature groups** | **Notes** | **References** |
| --- | --- | --- | --- | --- | --- |
| *LMNA*  (lamin A/C) | HGPS  (Hutchinson-Gilford progeria syndrome) | / | growth; head & neck; cardiovascular; chest; skeletal; skin, nails & hair; muscle, soft tissues; neurologic; metabolic features; hematology | / | (Carrero et al., 2016; Rieckher et al., 2021; Koschitzki et al., 2023; Coppedè, 2021; Worm et al., 2024; Schnabel et al., 2021; Navarro et al., 2006; Dreesen, 2020; Burla et al., 2018; Kubben and Misteli, 2017; Marbach et al., 2019; Marcelot et al., 2021; Elouej et al., 2020; Fu et al., 2024; Garg et al., 2022; Pignolo et al., 2020; Hisama et al., 2016; Lessel and Kubisch, 2019; Martin and Oshima, 2000; Hennekam, 2020; Lessel et al., 2014; Foo et al., 2019; Ghosh and Zhou, 2014; Milosic et al., 2024; OMIM) |
| *LMNA*  (lamin A/C) | APSs/AWS  (atypical progeroid syndromes / atypical Werner syndrome) | / | NA | / | (Carrero et al., 2016; Rieckher et al., 2021; Coppedè, 2021; Lessel et al., 2014; Burla et al., 2018; Marcelot et al., 2021; Garg et al., 2022; Hisama et al., 2016; Foo et al., 2019; Ghosh and Zhou, 2014; Milosic et al., 2024) |
| *ZMPSTE24*  (zinc metallopeptidase STE24)  alias symbol *FACE-1* |  |  |  |  | (Coppedè, 2021; Barrowman et al., 2012) |
| *LMNA*  (lamin A/C) | MAD  (mandibuloacral dysplasia) | MADA  (MAD with type A lipodystrophy) | growth; head & neck; chest; skeletal; skin, nails & hair; muscle, soft tissues; endocrine features | / | (Carrero et al., 2016; Rieckher et al., 2021; Koschitzki et al., 2023; Coppedè, 2021; Schnabel et al., 2021; Navarro et al., 2006; Dreesen, 2020; Burla et al., 2018; Marcelot et al., 2021; Elouej et al., 2020; Fu et al., 2024; Garg et al., 2022; Hennekam, 2020; Foo et al., 2019; Milosic et al., 2024; OMIM) |
| *ZMPSTE24*  (zinc metallopeptidase STE24)  alias symbol *FACE-1* |  | MADB  (MAD with type B lipodystrophy) | head & neck; chest; genitourinary; skeletal; skin, nails & hair; muscle, soft tissues; endocrine features |  | (Carrero et al., 2016; Rieckher et al., 2021; Koschitzki et al., 2023; Coppedè, 2021; Barrowman et al., 2012; Schnabel et al., 2021; Navarro et al., 2006; Dreesen, 2020; Burla et al., 2018; Marcelot et al., 2021; Elouej et al., 2020; Fu et al., 2024; Garg et al., 2022; Foo et al., 2019; Milosic et al., 2024; OMIM) |
| *MTX2*  (metaxin 2) | MADaM/MDPS  (mandibuloacral dysplasia associated to MTX2 / mandibuloacral dysplasia progeroid syndrome) | / | growth; head & neck; cardiovascular; respiratory; abdomen; genitourinary; skeletal; skin, nails & hair; muscle, soft tissues; neurologic | / | (Coppedè, 2021; Elouej et al., 2020; Fu et al., 2024; Garg et al., 2022; OMIM) |
| *POLD1*  (DNA polymerase delta 1, catalytic subunit) | MDPL  (mandibular hypoplasia, deafness, progeroid features, and lipodystrophy syndrome) | / | growth; head & neck; chest; abdomen; genitourinary; skeletal; skin, nails & hair; muscle, soft tissues; voice; endocrine features | / | (Coppedè, 2021; Worm et al., 2024; Schnabel et al., 2021; Burla et al., 2018; Elouej et al., 2020; Fu et al., 2024; Garg et al., 2022; Hisama et al., 2016; Lessel and Kubisch, 2019; Hennekam, 2020; OMIM) |
| *ZMPSTE24*  (zinc metallopeptidase STE24)  alias symbol *FACE-1* | RD/RSDM  (restrictive dermopathy) | RSDM1  (RSDM 1) | growth; head & neck; cardiovascular; respiratory; chest; genitourinary; skeletal; skin, nails & hair; endocrine features; prenatal manifestations | / | (Carrero et al., 2016; Rieckher et al., 2021; Koschitzki et al., 2023; Coppedè, 2021; Barrowman et al., 2012; Schnabel et al., 2021; Navarro et al., 2006; Dreesen, 2020; Burla et al., 2018; Marcelot et al., 2021; Hisama et al., 2016; Foo et al., 2019; Ghosh and Zhou, 2014; Milosic et al., 2024; OMIM) |
| *LMNA*  (lamin A/C) |  | RSDM2  (RSDM 2) | growth; head & neck; respiratory; chest; abdomen; genitourinary; skeletal; skin, nails & hair; prenatal manifestations |  | (Rieckher et al., 2021; Koschitzki et al., 2023; Coppedè, 2021; Schnabel et al., 2021; Navarro et al., 2006; Foo et al., 2019; Ghosh and Zhou, 2014; OMIM) |
| *BANF1*  (barrier to autointegration nuclear assembly factor 1)  alias symbol *BAF* | NGPS  (Néstor-Guillermo progeria syndrome) | / | growth; head & neck; cardiovascular; respiratory; chest; skeletal; skin, nails & hair; muscle, soft tissues | / | (Carrero et al., 2016; Rieckher et al., 2021; Koschitzki et al., 2023; Worm et al., 2024; Coppedè, 2021; Schnabel et al., 2021; Burla et al., 2018; Kubben and Misteli, 2017; Marbach et al., 2019; Marcelot et al., 2021; Elouej et al., 2020; Fu et al., 2024; Hennekam, 2020; Lessel et al., 2014; Foo et al., 2019; Milosic et al., 2024; OMIM) |
| *WRN*  (WRN RecQ like helicase)  alias symbols *RECQL2* and *RECQ3* | WS  (Werner syndrome) | / | growth; head & neck; cardiovascular; skeletal; skin, nails & hair; endocrine features; neoplasia | / | (Carrero et al., 2016; Rieckher et al., 2021; Koschitzki et al., 2023; Worm et al., 2024; Coppedè, 2021; Schnabel et al., 2021; Navarro et al., 2006; Burla et al., 2018; Kubben and Misteli, 2017; Abu-Libdeh et al., 2022; Pignolo et al., 2020; Hisama et al., 2016; Lessel and Kubisch, 2019; Lessel et al., 2014; Martin and Oshima, 2000; Hennekam, 2020; Milosic et al., 2024; OMIM) |
| *BLM*  (BLM RecQ like helicase)  alias symbols *BS*, *RECQL3* and *RECQ2* | BS  (Bloom syndrome) | / | growth; head & neck; respiratory; genitourinary; skeletal; skin, nails & hair; neurologic; voice; endocrine features; immunology; neoplasia | / | (Carrero et al., 2016; Rieckher et al., 2021; Koschitzki et al., 2023; Worm et al., 2024; Coppedè, 2021; Schnabel et al., 2021; Navarro et al., 2006; Burla et al., 2018; Kubben and Misteli, 2017; Abu-Libdeh et al., 2022; Hisama et al., 2016; Hennekam, 2020; Lessel et al., 2014; Martin et al., 2018; Milosic et al., 2024; OMIM) |
| *ANAPC1*  (anaphase promoting complex subunit 1) | RTS  (Rothmund-Thomson  syndrome) | RTS1  (RTS, type 1) | growth; head & neck; genitourinary; skeletal; skin, nails & hair; neurologic; endocrine features | / | (Schnabel et al., 2021; Martins et al., 2023; Di Lazzaro Filho et al., 2023; Averdunk et al., 2023; OMIM) |
| *RECQL4*  (RecQ like helicase 4)  alias symbol *RECQ4* |  | RTS2  (RTS, type 2) | growth; head & neck; abdomen; genitourinary; skeletal; skin, nails & hair; neurologic; endocrine features; neoplasia |  | (Carrero et al., 2016; Rieckher et al., 2021; Koschitzki et al., 2023; Worm et al., 2024; Coppedè, 2021; Schnabel et al., 2021; Navarro et al., 2006; Burla et al., 2018; Abu-Libdeh et al., 2022; Martins et al., 2023; Di Lazzaro Filho et al., 2023; Averdunk et al., 2023; Hisama et al., 2016; Martin and Oshima, 2000; Milosic et al., 2024; OMIM) |
| *CRIPT*  (CXXC repeat containing interactor of PDZ3 domain) |  | RTS3  (RTS, type 3) | growth; head & neck; cardiovascular; respiratory; chest; skeletal; skin, nails & hair; neurologic; hematology; immunology |  | (OMIM; Averdunk et al., 2023; Martins et al., 2023) |
| *DNA2*  (DNA replication helicase/nuclease 2) |  | RTS4  (RTS, type 4) | growth; head & neck; genitourinary; skeletal; skin, nails & hair; neurologic; endocrine features |  | (OMIM; Di Lazzaro Filho et al., 2023; Martins et al., 2023) |
| *ERCC8*  (ERCC excision repair 8, CSA ubiquitin ligase complex subunit)  alias symbol *CSA*; previous symbol *CKN1* | CS  (Cockayne syndrome) | CSA  (CS, type A) | growth; head & neck; cardiovascular; abdomen; genitourinary; skeletal; skin, nails & hair; muscle, soft tissues; neurologic; endocrine features | / | (Carrero et al., 2016; Rieckher et al., 2021; Koschitzki et al., 2023; Schnabel et al., 2021; Navarro et al., 2006; Burla et al., 2018; Kubben and Misteli, 2017; Pignolo et al., 2020; Hisama et al., 2016; Martin and Oshima, 2000; Hennekam, 2020; Milosic et al., 2024; Natale and Raquer, 2017; Ferri et al., 2020; Kraemer et al., 2007; OMIM) |
| *ERCC6*  (ERCC excision repair 6, chromatin remodeling factor)  alias symbol *CSB*; previous symbol *CKN2* |  | CSB  (CS, type B) | growth; head & neck; cardiovascular; abdomen; genitourinary; skeletal; skin, nails & hair; muscle, soft tissues; neurologic |  | (Carrero et al., 2016; Rieckher et al., 2021; Koschitzki et al., 2023; Worm et al., 2024; Schnabel et al., 2021; Navarro et al., 2006; Burla et al., 2018; Kubben and Misteli, 2017; Pignolo et al., 2020; Hisama et al., 2016; Martin and Oshima, 2000; Hennekam, 2020; Milosic et al., 2024; Ferri et al., 2020; Natale and Raquer, 2017; Colella et al., 2000; Kraemer et al., 2007; OMIM) |
| *ERCC6*  (ERCC excision repair 6, chromatin remodeling factor)  alias symbol *CSB*; previous symbol *CKN2* | COFS  (cerebro-oculo-facio-skeletal syndrome) | COFS1  (COFS 1) | growth; head & neck; chest; skeletal; skin, nails & hair; neurologic | COFS is also considered a severe form of CS (Cockayne syndrome) (Ferri et al., 2020; Kraemer et al., 2007). | (Schnabel et al., 2021; Ferri et al., 2020; Kraemer et al., 2007; Gregg et al., 2011; OMIM) |
| *ERCC2*  (ERCC excision repair 2, TFIIH core complex helicase subunit gene)  previous symbol *XPD* |  | COFS2  (COFS 2) | growth; head & neck; respiratory; genitourinary; skeletal; skin, nails & hair; neurologic | OMIM indicataes a provisional relationship between *ERCC2* and COFS2. | (Ferri et al., 2020; Kraemer et al., 2007; Gregg et al., 2011; OMIM) |
| *ERCC5*  (ERCC excision repair 5, endonuclease)  previous symbol *XPGC*; also referred to as *XPG* (symbol not available in HGNC) |  | COFS3  (COFS 3) | growth; head & neck; skeletal; skin, nails & hair; muscle, soft tissues; neurologic; prenatal manifestations | / | (Ferri et al., 2020; Gregg et al., 2011; OMIM) |
| *ERCC1*  (ERCC excision repair 1, endonuclease non-catalytic subunit) |  | COFS4  (COFS 4) | growth; head & neck; skeletal; neurologic |  | (Ferri et al., 2020; Gregg et al., 2011; OMIM) |
| *XPA*  (XPA, DNA damage recognition and repair factor) | XP  (xeroderma pigmentosum) | XPA  (XP, complementation group A) | head & neck; skin, nails & hair; neurologic | Pathogenic variants in genes *ERCC2* (*XPD*), *ERCC3* (*XPB*), *ERCC4* (*XPF*), *ERCC5* (*XPG*) and *ERCC1* can also result in rare combinations of XP with CS (XP/CS complex), where patients display a combination of XP and CS clinical features (Ferri et al., 2020; Natale and Raquer, 2017; Dubaele et al., 2003; Colella et al., 2000; Koschitzki et al., 2023; Rieckher et al., 2021; Hisama et al., 2016; OMIM). | (Carrero et al., 2016; Koschitzki et al., 2023; Worm et al., 2024; Schnabel et al., 2021; Burla et al., 2018; Kubben and Misteli, 2017; Milosic et al., 2024; Natale and Raquer, 2017; Ferri et al., 2020; Kraemer et al., 2007; OMIM) |
| *ERCC3*  (ERCC excision repair 3, TFIIH core complex helicase subunit)  alias symbol *XPB* |  | XPB  (XP, complementation group B) | growth; head & neck; skin, nails & hair; neurologic; endocrine features; neoplasia |  | (Carrero et al., 2016; Koschitzki et al., 2023; Worm et al., 2024; Schnabel et al., 2021; Kubben and Misteli, 2017; Milosic et al., 2024; Natale and Raquer, 2017; Ferri et al., 2020; Kraemer et al., 2007; OMIM) |
| *XPC*  (XPC complex subunit, DNA damage recognition and repair factor) |  | XPC  (XP, complementation group C) | head & neck; skin, nails & hair |  | (Carrero et al., 2016; Koschitzki et al., 2023; Worm et al., 2024; Schnabel et al., 2021; Kubben and Misteli, 2017; Milosic et al., 2024; Natale and Raquer, 2017; Ferri et al., 2020; Kraemer et al., 2007; OMIM) |
| *ERCC2*  (ERCC excision repair 2, TFIIH core complex helicase subunit gene)  previous symbol *XPD* |  | XPD  (XP, complementation group D) | head & neck; skin, nails & hair; neurologic; neoplasia |  | (Koschitzki et al., 2023; Schnabel et al., 2021; Kubben and Misteli, 2017; Milosic et al., 2024; Natale and Raquer, 2017; Ferri et al., 2020; Dubaele et al., 2003; Kraemer et al., 2007; OMIM) |
| *DDB2*  (damage specific DNA binding protein 2)  alias symbol *XPE* |  | XPE  (XP, complementation group E) | head & neck; skin, nails & hair |  | (Carrero et al., 2016; Koschitzki et al., 2023; Worm et al., 2024; Schnabel et al., 2021; Kubben and Misteli, 2017; Milosic et al., 2024; Natale and Raquer, 2017; Ferri et al., 2020; Kraemer et al., 2007; OMIM) |
| *ERCC4*  (ERCC excision repair 4, endonuclease catalytic subunit)  previous symbol *XPF*; alias symbol *FANCQ* |  | XPF  (XP, complementation group F) | growth; head & neck; skeletal; skin, nails & hair; neurologic; neoplasia |  | (Carrero et al., 2016; Koschitzki et al., 2023; Worm et al., 2024; Schnabel et al., 2021; Kubben and Misteli, 2017; Milosic et al., 2024; Natale and Raquer, 2017; Ferri et al., 2020; Kraemer et al., 2007; Gregg et al., 2011; OMIM) |
| *ERCC5*  (ERCC excision repair 5, endonuclease)  previous symbol *XPGC*; also referred to as *XPG* (symbol not available in HGNC) |  | XPG  (XP, complementation group G) | growth; head & neck; skeletal; skin, nails & hair; neurologic |  | (Carrero et al., 2016; Koschitzki et al., 2023; Worm et al., 2024; Schnabel et al., 2021; Navarro et al., 2006; Kubben and Misteli, 2017; Milosic et al., 2024; Natale and Raquer, 2017; Ferri et al., 2020; Kraemer et al., 2007; OMIM) |
| *POLH*  (DNA polymerase eta)  alias symbol *XPV* |  | XPV  (XP, complementation group V) | head & neck; skin, nails & hair |  | (Carrero et al., 2016; Worm et al., 2024; Schnabel et al., 2021; Kubben and Misteli, 2017; Milosic et al., 2024; Natale and Raquer, 2017; Kraemer et al., 2007; OMIM) |
| *ERCC6*  (ERCC excision repair 6, chromatin remodeling factor)  alias symbol *CSB*; previous symbol *CKN2* |  | NA | NA |  | (Carrero et al., 2016; Kubben and Misteli, 2017) |
| *ERCC6*  (ERCC excision repair 6, chromatin remodeling factor)  alias symbol *CSB*; previous symbol *CKN2* | DSC  (DeSanctis-Cacchione syndrome) | / | growth; head & neck; skeletal; skin, nails & hair; neurologic | DSC is also considered a form/variant of XP (xeroderma pigmentosum) with a more severe clinical phenotype (Colella et al., 2000; OMIM). According to OMIM, the clinical entity of DSC can manifest in patients with various forms of XP, although it is most commonly associated with complementation group A (XPA).  OMIM indicates a provisional relationship between *ERCC6* and DSC. | (Rieckher et al., 2021; Koschitzki et al., 2023; Schnabel et al., 2021; Colella et al., 2000; OMIM) |
| *ERCC2*  (ERCC excision repair 2, TFIIH core complex helicase subunit gene)  previous symbol *XPD* | TTD  (trichothiodystrophy) | TTD1  (TTD 1, photosensitive) | growth; head & neck; abdomen; skin, nails & hair; neurologic; immunology; neoplasia | Pathogenic variants in the *ERCC2* (*XPD*) gene can also result in rare combinations of XP with TTD (XP/TTD complex), showing combined features of both diseases (Ferri et al., 2020; Kraemer et al., 2007; Dubaele et al., 2003). | (Carrero et al., 2016; Rieckher et al., 2021; Koschitzki et al., 2023; Kubben and Misteli, 2017; Milosic et al., 2024; Ferri et al., 2020; Dubaele et al., 2003; Kraemer et al., 2007; OMIM) |
| *ERCC3*  (ERCC excision repair 3, TFIIH core complex helicase subunit)  alias symbol *XPB* |  | TTD2  (TTD 2, photosensitive) | growth; head & neck; skin, nails & hair; neurologic | / | (Carrero et al., 2016; Rieckher et al., 2021; Koschitzki et al., 2023; Kubben and Misteli, 2017; Milosic et al., 2024; Ferri et al., 2020; Kraemer et al., 2007; OMIM) |
| *GTF2H5*  (general transcription factor IIH subunit 5)  alias symbols *TFB5* and *TTDA* |  | TTD3  (TTD 3, photosensitive) | growth; head & neck; skin, nails & hair; neurologic; immunology; prenatal manifestations |  | (Carrero et al., 2016; Rieckher et al., 2021; Koschitzki et al., 2023; Kubben and Misteli, 2017; Milosic et al., 2024; Ferri et al., 2020; Kraemer et al., 2007; OMIM) |
| *MPLKIP*  (M-phase specific PLK1 interacting protein)  previous symbol *C7orf11*; alias symbol *TTDN1* |  | TTD4  (TTD 4, nonphotosensitive) | growth; head & neck; cardiovascular; genitourinary; skeletal; skin, nails & hair; neurologic |  | (Koschitzki et al., 2023; Milosic et al., 2024; Kraemer et al., 2007; OMIM) |
| *RNF113A*  (ring finger protein 113A ) |  | TTD5  (TTD 5, nonphotosensitive) | growth; head & neck; abdomen; genitourinary; skeletal; skin, nails & hair; muscle, soft tissues; neurologic; endocrine features; hematology; immunology |  | (Koschitzki et al., 2023; OMIM) |
| *GTF2E2*  (general transcription factor IIE subunit 2)  alias symbol *TFIIE-B* |  | TTD6  (TTD 6, nonphotosensitive) | growth; head & neck; skeletal; skin, nails & hair; neurologic; hematology |  | (Koschitzki et al., 2023; OMIM) |
| *TARS1*  (threonyl-tRNA synthetase 1)  previous symbol *TARS* |  | TTD7  (TTD 7, nonphotosensitive) | skin, nails & hair |  | (Koschitzki et al., 2023; OMIM) |
| *AARS1*  (alanyl-tRNA synthetase 1) |  | TTD8  (TTD 8, nonphotosensitive) | growth; head & neck; skin, nails & hair; neurologic; endocrine features |  | (Koschitzki et al., 2023; OMIM) |
| *MARS1*  (methionyl-tRNA synthetase 1) |  | TTD9  (TTD 9, nonphotosensitive) | growth; head & neck; genitourinary; skeletal; skin, nails & hair; neurologic; endocrine features | OMIM indicates a provisional relationship between *MARS1* and TTD9. | (Koschitzki et al., 2023; OMIM) |
| *FANCA*  (FA complementation group A)  previous symbol *FANCH* | FA  (Fanconi anemia) | FANCA  (FA, complementation group A) | growth; head & neck; cardiovascular; genitourinary; skeletal; skin, nails & hair; neurologic; endocrine features; hematology | A previously designated Fanconi anemia complementation group (FANCH) was later determined to represent the same clinical entity as FANCA and is therefore no longer recognized as a distinct group (OMIM). | (Carrero et al., 2016; Koschitzki et al., 2023; Worm et al., 2024; Milosic et al., 2024; OMIM) |
| *FANCB*  (FA complementation group B) |  | FANCB  (FA, complementation group B) | growth; head & neck; genitourinary; skeletal; skin, nails & hair; neurologic; endocrine features; hematology | / | (Carrero et al., 2016; Koschitzki et al., 2023; Milosic et al., 2024; OMIM) |
| *FANCC*  (FA complementation group C) |  | FANCC  (FA, complementation group C) | growth; head & neck; skeletal; hematology; skin, nails & hair; cardiovascular; genitourinary; neurologic |  | (Carrero et al., 2016; Koschitzki et al., 2023; Milosic et al., 2024; OMIM) |
| *BRCA2*  (BRCA2 DNA repair associated)  previous symbols *FANCD1*, *FANCD*, *FACD*; alias symbol *XRCC11* |  | FANCD1  (FA, complementation  group D1) | growth; head & neck; cardiovascular; abdomen; genitourinary; skeletal; skin, nails & hair; hematology; neoplasia |  | (Carrero et al., 2016; Koschitzki et al., 2023; Milosic et al., 2024; OMIM) |
| *FANCD2*  (FA complementation group D2) |  | FANCD2  (FA, complementation group D2) | growth; head & neck; cardiovascular; genitourinary; skeletal; skin, nails & hair; neurologic; hematology; neoplasia |  | (Carrero et al., 2016; Koschitzki et al., 2023; Milosic et al., 2024; OMIM) |
| *FANCE*  (FA complementation group E) |  | FANCE  (FA, complementation group E) | growth; head & neck; cardiovascular; genitourinary; skeletal; skin, nails & hair; neurologic; endocrine features; hematology |  | (Carrero et al., 2016; Koschitzki et al., 2023; Milosic et al., 2024; OMIM) |
| *FANCF*  (FA complementation group F) |  | FANCF  (FA, complementation group F) | growth; head & neck; cardiovascular; genitourinary; skeletal; skin, nails & hair; endocrine features; hematology; prenatal manifestations |  | (Carrero et al., 2016; Koschitzki et al., 2023; Milosic et al., 2024; OMIM) |
| *FANCG*  (FA complementation group G)  previous symbol *XRCC9* |  | FANCG  (FA, complementation group G) | growth; head & neck; skeletal; skin, nails & hair; hematology |  | (Carrero et al., 2016; Koschitzki et al., 2023; Milosic et al., 2024; OMIM) |
| *FANCI*  (FA complementation group I) |  | FANCI  (FA, complementation group I) | growth; head & neck; cardiovascular; abdomen; genitourinary; skeletal; skin, nails & hair; neurologic; endocrine features; hematology |  | (Carrero et al., 2016; Koschitzki et al., 2023; Milosic et al., 2024; OMIM) |
| *BRIP1*  (BRCA1 interacting DNA helicase 1)  alias symbol *FANCJ* |  | FANCJ  (FA, complementation group J) | NA |  | (Carrero et al., 2016; Koschitzki et al., 2023; Milosic et al., 2024; OMIM) |
| *FANCL*  (FA complementation group L)  previous symbol *PHF9* |  | FANCL  (FA, complementation group L) | growth; head & neck; cardiovascular; respiratory; abdomen; genitourinary; skeletal; neurologic; hematology |  | (Carrero et al., 2016; Koschitzki et al., 2023; Milosic et al., 2024; OMIM) |
| *FANCM*  (FA complementation group M)  alias symbol *FAAP250* |  | FANCM  (FA, complementation group M) | NA | A clinical case initially attributed to a pathogenic variant in the *FAAP250* (*FANCM*) gene (originally designated as FANCM) was subsequently re-evaluated and reclassified as FANCA (OMIM). | (Carrero et al., 2016; Koschitzki et al., 2023; Milosic et al., 2024) |
| *PALB2*  (partner and localizer of BRCA2)  alias symbol *FANCN* |  | FANCN  (FA, complementation group N) | growth; head & neck; cardiovascular; abdomen; genitourinary; skeletal; skin, nails & hair; hematology; neoplasia |  | (Carrero et al., 2016; Koschitzki et al., 2023; Milosic et al., 2024; OMIM) |
| *RAD51C*  (RAD51 paralog C)  alias symbol *FANCO* |  | FANCO  (FA, complementation group O) | growth; cardiovascular; abdomen; genitourinary; skeletal |  | (Carrero et al., 2016; Koschitzki et al., 2023; Milosic et al., 2024; OMIM) |
| *SLX4*  (SLX4 structure-specific endonuclease subunit)  alias symbol *FANCP* |  | FANCP  (FA, complementation group P) | growth; head & neck; genitourinary; skeletal; skin, nails & hair; hematology; neoplasia |  | (Carrero et al., 2016; Koschitzki et al., 2023; Milosic et al., 2024; OMIM) |
| *ERCC4*  (ERCC excision repair 4, endonuclease catalytic subunit)  previous symbol *XPF*; alias symbol *FANCQ* |  | FANCQ  (FA, complementation group Q) | growth; head & neck; abdomen; skeletal; hematology |  | (Koschitzki et al., 2023; Ferri et al., 2020; OMIM) |
| *RAD51*  (RAD51 recombinase)  alias symbol *FANCR* |  | FANCR  (FA, complementation group R) | growth; head & neck; abdomen; genitourinary; skeletal; neurologic; hematology |  | (Koschitzki et al., 2023; OMIM) |
| *BRCA1*  (BRCA1 DNA repair associated)  alias symbol *FANCS* |  | FANCS  (FA, complementation group S) | growth; head & neck; skeletal; skin, nails & hair; neurologic; hematology; neoplasia |  | (Koschitzki et al., 2023; OMIM) |
| *UBE2T*  (ubiquitin conjugating enzyme E2 T)  alias symbol *FANCT* |  | FANCT  (FA, complementation group T) | growth; skeletal; hematology; neoplasia |  | (Koschitzki et al., 2023; OMIM) |
| *XRCC2*  (X-ray repair cross complementing 2)  alias symbol *FANCU* |  | FANCU  (FA, complementation group U) | growth; head & neck; cardiovascular; genitourinary; skeletal; hematology | OMIM indicates a provisional relationship between *XRCC2* and FANCU. | (Koschitzki et al., 2023; OMIM) |
| *MAD2L2*  (mitotic arrest deficient 2 like 2)  alias symbols *REV7* and *FANCV* |  | FANCV  (FA, complementation group V) | growth; head & neck; genitourinary; hematology | OMIM indicates a provisional relationship between *MAD2L2* and FANCV. | (Koschitzki et al., 2023; OMIM) |
| *RFWD3*  (ring finger and WD repeat domain 3)  alias symbol *FANCW* |  | FANCW  (FA, complementation group W) | growth; head & neck; abdomen; genitourinary; skeletal; neurologic; hematology | OMIM indicates a provisional relationship between *RFWD3* and FANCW. | (Koschitzki et al., 2023; OMIM) |
| *ERCC4*  (ERCC excision repair 4, endonuclease catalytic subunit)  previous symbol *XPF*; alias symbol *FANCQ* | XFEPS  (XFE progeroid syndrome) | / | growth; head & neck; cardiovascular; abdomen; genitourinary; skeletal; skin, nails & hair; muscle, soft tissues; neurologic; voice; neoplasia | / | (Rieckher et al., 2021; Ferri et al., 2020; Gregg et al., 2011; OMIM) |
| *ERCC1*  (ERCC excision repair 1, endonuclease non-catalytic subunit) |  |  |  | *ERCC1* forms a functional complex with *ERCC4* (*XPF*). While pathogenic variants in *ERCC4* underlie XFE progeroid syndrome in humans, *ERCC1* is essential for complex activity, and its deficiency produces XFEPS-like phenotypes in murine models (Gregg et al., 2011; Rieckher et al., 2021; OMIM). | (Rieckher et al., 2021; Gregg et al., 2011; OMIM) |
| *ATR*  (ATR serine/threonine kinase) | SS/SCKL  (Seckel syndrome) | SCKL1  (SCKL 1) | growth; head & neck; chest; genitourinary; skeletal; skin, nails & hair; neurologic; hematology | A previously reported Seckel syndrome locus on chromosome 14q (designated SCKL3) was later reconsidered (OMIM). | (Carrero et al., 2016; Worm et al., 2024; Rieckher et al., 2021; Schnabel et al., 2021; Ogi et al., 2012; Yigit et al., 2015; Griffith et al., 2008; Willems et al., 2010; OMIM) |
| *RBBP8*  (RB binding protein 8, endonuclease)  alias symbol *CtIP* |  | SCKL2  (SCKL 2) | growth; head & neck; cardiovascular; genitourinary; skeletal; skin, nails & hair; neurologic; voice |  | (Worm et al., 2024; Schnabel et al., 2021; Ogi et al., 2012; Yigit et al., 2015; OMIM) |
| *CPAP*  (centrosome assembly and centriole elongation protein)  previous symbol *CENPJ* |  | SCKL4  (SCKL 4) | growth; head & neck; chest; skeletal; neurologic | OMIM indicates a provisional relationship between *CPAP* and SCKL4. | (Schnabel et al., 2021; Ogi et al., 2012; Yigit et al., 2015; Shaheen et al., 2014; OMIM) |
| *CEP152*  (centrosomal protein 152) |  | SCKL5  (SCKL 5) | growth; head & neck; chest; genitourinary; skeletal; neurologic | / | (Schnabel et al., 2021; Ogi et al., 2012; Yigit et al., 2015; Shaheen et al., 2014; OMIM) |
| *CEP63*  (centrosomal protein 63) |  | SCKL6  (SCKL 6) | growth; head & neck; neurologic | OMIM indicates a provisional relationship the *CEP63* and SCKL6. | (Schnabel et al., 2021; Yigit et al., 2015; OMIM) |
| *NIN*  (ninein) |  | SCKL7  (SCKL 7) | growth; head & neck; chest; genitourinary; skeletal; neurologic; endocrine features | OMIM indicates a provisional relationship between *NIN* and SCKL7. | (Schnabel et al., 2021; Yigit et al., 2015; OMIM) |
| *DNA2*  (DNA replication helicase/nucelase 2) |  | SCKL8  (SCKL 8) | growth; head & neck; genitourinary; skeletal; neurologic | / | (Schnabel et al., 2021; Yigit et al., 2015; OMIM) |
| *TRAIP*  (TRAF interacting protein) |  | SCKL9  (SCKL 9) | growth; head & neck; cardiovascular; respiratory; chest; genitourinary; skeletal; skin, nails & hair; neurologic; prenatal manifestations |  | (Schnabel et al., 2021; OMIM) |
| *NSMCE2*  (NSE2 (MMS21) homolog, SMC5-SMC6 complex SUMO ligase) |  | SCKL10  (SCKL 10) | growth; head & neck; cardiovascular; abdomen; genitourinary; skeletal; skin, nails & hair; muscle, soft tissues; neurologic; metabolic features; endocrine features |  | (Schnabel et al., 2021; OMIM) |
| *ATRIP*  (ATR interacting protein) |  | NA | NA |  | (Schnabel et al., 2021; Ogi et al., 2012; Yigit et al., 2015) |
| *CDK5RAP2*  (CDK5 regulatory subunit associated protein 2) |  | NA | NA | Phenotypic overlaps of SCKL with other disorders characterized by microcephaly and/or primordial dwarfism can lead to variations in gene-syndrome associations and classification and diagnostic challenges (Ogi et al., 2012; Willems et al., 2010; OMIM). | (Schnabel et al., 2021; Yigit et al., 2015) |
| *PCNT*  (pericentrin)  alias symbol *SCKL4* |  | NA | NA |  | (Griffith et al., 2008; Willems et al., 2010) |
| *PLK4*  (polo like kinase 4) |  | NA | NA |  | (Schnabel et al., 2021; Martin et al., 2014; Shaheen et al., 2014) |
| *ATM*  (ATM serine/threonine kinase) | AT  (ataxia  telangiectasia)  = Louis-Bar syndrome | / | growth; head & neck; respiratory; genitourinary; skin, nails & hair; neurologic; endocrine features; immunology; neoplasia | / | (Carrero et al., 2016; Rieckher et al., 2021; Koschitzki et al., 2023; Coppedè, 2021; Worm et al., 2024; Schnabel et al., 2021; Kubben and Misteli, 2017; Marcelot et al., 2021; Pignolo et al., 2020; Hisama et al., 2016; Martin and Oshima, 2000; Milosic et al., 2024; OMIM) |
| *MRE11*  (MRE11 homolog, double strand break repair nuclease)  previous symbol *MRE11A*; alias symbol *ATLD* | ATLD  (ataxia telangiectasia-like disorder) | ATLD1  (ATLD 1) | head & neck; muscle, soft tissues; neurologic | / | (Carrero et al., 2016; OMIM) |
| *PCNA*  (proliferating cell nuclear antigen) |  | ATLD2  (ATLD 2) | growth; head & neck; abdomen; skeletal; skin, nails & hair; muscle, soft tissues; neurologic; neoplasia | OMIM indicates a provisional relationship between *PCNA*  and ATLD2. | (OMIM; Baple et al., 2014) |
| *CTC1*  (CST telomere replication complex component 1) | CRMCC  (cerebroretinal microangiopathy with calcifications and cysts) = Coats plus syndrome | CRMCC1 (CRMCC 1) | growth; head & neck; cardiovascular; abdomen; skeletal; skin, nails & hair; neurologic; hematology | / | (Carrero et al., 2016; Rieckher et al., 2021; Simon et al., 2016; Takai et al., 2016; OMIM) |
| *STN1*  (STN1 subunit of CST complex) |  | CRMCC2 (CRMCC 2) | growth; head & neck; cardiovascular; abdomen; skeletal; skin, nails & hair; neurologic; hematology |  | (OMIM; Simon et al., 2016) |
| *POT1*  (protection of telomeres 1) |  | CRMCC3 (CRMCC 3) | growth; head & neck; abdomen; skeletal; skin, nails & hair; neurologic | OMIM indicates a provisional relationship between *POT1* and CRMCC3. | (OMIM; Takai et al., 2016) |
| *TERC*  (telomerase RNA component) | DC/DKC  (dyskeratosis congenita) | DKCA1  (DKC, autosomal dominant 1) | head & neck; respiratory; abdomen; skin, nails & hair; hematology; neoplasia | DKCA are autosomal dominant forms, DKCB are autosomal recessive forms. DKCX is X-linked recessive form (OMIM). | (Carrero et al., 2016; Rieckher et al., 2021; Koschitzki et al., 2023; Worm et al., 2024; Schnabel et al., 2021; Burla et al., 2018; Kubben and Misteli, 2017; Pignolo et al., 2020; Hennekam, 2020; Milosic et al., 2024; Kilic and Cekic, 2016; Walne et al., 2010; OMIM) |
| *TERT*  (telomerase reverse transcriptase) |  | DKCA2  (DKC, autosomal dominant 2) | growth; head & neck; cardiovascular; respiratory; abdomen; genitourinary; skeletal; skin, nails & hair; neurologic; hematology; immunology |  | (Carrero et al., 2016; Rieckher et al., 2021; Koschitzki et al., 2023; Schnabel et al., 2021; Burla et al., 2018; Kubben and Misteli, 2017; Milosic et al., 2024; Kilic and Cekic, 2016; Walne et al., 2010; OMIM) |
| *TERT*  (telomerase reverse transcriptase) |  | DKCB4  (DKC, autosomal recessive 4) | growth; head & neck; cardiovascular; respiratory; abdomen; genitourinary; skeletal; skin, nails & hair; hematology; immunology |  | (Carrero et al., 2016; Rieckher et al., 2021; Koschitzki et al., 2023; Schnabel et al., 2021; Burla et al., 2018; Kubben and Misteli, 2017; Milosic et al., 2024; Kilic and Cekic, 2016; Walne et al., 2010; OMIM) |
| *WRAP53*  (WD repeat containing protein antisense to TP53)  alias symbol *TCAB1* |  | DKCB3  (DKC, autosomal recessive 3) | head & neck; skin, nails & hair; hematology; neoplasia |  | (Carrero et al., 2016; Koschitzki et al., 2023; Schnabel et al., 2021; Kubben and Misteli, 2017; Milosic et al., 2024; Kilic and Cekic, 2016; OMIM) |
| *CTC1*  (CST telomere replication complex component 1) |  | NA | NA | OMIM classifies *CTC1* pathogenic variants as causative for  cerebroretinal microangiopathy with calcifications and cysts (CRMCC1; see above), another telomere-related disorder with overlapping phenotypic features of DKC (OMIM). | (Carrero et al., 2016; Koschitzki et al., 2023; Schnabel et al., 2021; Kubben and Misteli, 2017; Milosic et al., 2024) |
| *NOP10*  (NOP10 ribonucleoprotein)  previous symbol *NOLA3* |  | DKCB1  (DKC, autosomal recessive 1) | head & neck; respiratory; abdomen; skeletal; skin, nails & hair; neurologic; hematology; neoplasia | OMIM indicates a provisional relationship between *NOP10* and DKCB1. | (Rieckher et al., 2021; Koschitzki et al., 2023; Schnabel et al., 2021; Hennekam, 2020; Milosic et al., 2024; Kilic and Cekic, 2016; Walne et al., 2010; OMIM) |
| *NHP2*  (NHP2 ribonucleoprotein)  previous symbol *NOLA2* |  | DKCB2  (DKC, autosomal recessive 2) | growth; head & neck; abdomen; genitourinary; skin, nails & hair; neurologic; hematology; immunology | / | (Rieckher et al., 2021; Koschitzki et al., 2023; Schnabel et al., 2021; Milosic et al., 2024; Kilic and Cekic, 2016; Walne et al., 2010; OMIM) |
| *RTEL1*  (regulator of telomere elongation helicase 1) |  | DKCA4  (DKC, autosomal dominant 4) | growth; head & neck; abdomen; skin, nails & hair; neurologic; hematology; immunology |  | (Rieckher et al., 2021; Koschitzki et al., 2023; Schnabel et al., 2021; Kilic and Cekic, 2016; OMIM) |
| *RTEL1*  (regulator of telomere elongation helicase 1) |  | DKCB5  (DKC, autosomal recessive 5) | growth; head & neck; abdomen; skin, nails & hair; neurologic; hematology; immunology |  | (Rieckher et al., 2021; Koschitzki et al., 2023; Schnabel et al., 2021; Kilic and Cekic, 2016; OMIM) |
| *PARN*  (poly(A)-specific ribonuclease) |  | DKCB6  (DKC, autosomal recessive 6) | growth; head & neck; skin, nails & hair; neurologic; hematology |  | (Rieckher et al., 2021; Schnabel et al., 2021; Milosic et al., 2024; OMIM) |
| *DKC1*  (dyskerin pseudouridine synthase 1)  previous symbol *DKC*; alias symbol *NOLA4* |  | DKCX  (DKC, X-linked) | growth; head & neck; respiratory; abdomen; genitourinary; skeletal; skin, nails & hair; muscle, soft tissues; neurologic; hematology; immunology; neoplasia |  | (Rieckher et al., 2021; Koschitzki et al., 2023; Schnabel et al., 2021; Burla et al., 2018; Pignolo et al., 2020; Hennekam, 2020; Milosic et al., 2024; Kilic and Cekic, 2016; Walne et al., 2010; OMIM) |
| *TINF2*  (TERF1 interacting nuclear factor 2) |  | DKCA3  (DKC, autosomal dominant 3) | growth; head & neck; respiratory; abdomen; genitourinary; skeletal; skin, nails & hair; neurologic; hematology; neoplasia |  | (Koschitzki et al., 2023; Schnabel et al., 2021; Kilic and Cekic, 2016; Walne et al., 2010; OMIM) |
| *TINF2*  (TERF1 interacting nuclear factor 2) |  | DKCA5  (DKC, autosomal dominant 5) = Revesz syndrome | growth; head & neck; skin, nails & hair; neurologic; hematology |  | (Koschitzki et al., 2023; Schnabel et al., 2021; Kilic and Cekic, 2016; Walne et al., 2010; OMIM) |
| *ACD*  (ACD shelterin complex subunit and telomerase recruitment factor)  alias symbol *TPP1* |  | DKCA6  (DKC, autosomal dominant 6) | growth; head & neck; abdomen; skin, nails & hair; neurologic; hematology; neoplasia | OMIM indicates a provisional relationship between *ACD* and DKCA6. | (Rieckher et al., 2021; Schnabel et al., 2021; OMIM) |
| *ACD*  (ACD shelterin complex subunit and telomerase recruitment factor)  alias symbol *TPP1* |  | DKCB7  (DKC, autosomal recessive 7) | growth; head & neck; abdomen; skin, nails & hair; neurologic; hematology; neoplasia | OMIM indicates a provisional relationship between *ACD* and DKCB7. | (Rieckher et al., 2021; Schnabel et al., 2021; OMIM) |
| *DCLRE1B*  (DNA cross-link repair 1B)  alias symbols *APPOLO* and *SNM1B* |  | DKCB8  (DKC, autosomal recessive 8) | growth; head & neck; abdomen; skin, nails & hair; neurologic; hematology; immunology |  | (Milosic et al., 2024; Kermasson et al., 2022; OMIM) |
| *TYMS*  (thymidylate synthetase) |  | DKCD  (DKC, digenic) | growth; head & neck; respiratory; skin, nails & hair; immunology | DKCD is a digenic form of disorder, caused by variants in the *TYMS* gene combined with variants in the *ENOSF1* gene (OMIM). | (OMIM; Tummala et al., 2022) |
| *ENOSF1*  (enolase superfamily member 1) |  |  |  |  | (OMIM; Tummala et al., 2022) |
| *USB1*  (U6 snRNA biogenesis phosphodiesterase 1)  previous symbol *C16orf57* |  | NA | NA | Phenotypic overlap between DKC and other inherited poikilodermas complicates classification and diagnosis (Walne et al., 2010). | (Koschitzki et al., 2023; Kilic and Cekic, 2016; Walne et al., 2010) |
| *DKC1*  (dyskerin pseudouridine synthase 1)  previous symbol *DKC*; alias symbol *NOLA4* | HHS  (Hoyeraal-Hreidarsson syndrome) | / | growth; head & neck; abdomen; neurologic; hematology; immunology | HHS is also considered a severe clinical variant of DKC, and can be caused by pathogenic variants in several different DKC-associated genes (Carrero et al., 2016; Glousker et al., 2015; Schnabel et al., 2021; OMIM). | (Glousker et al., 2015; OMIM) |
| *TINF2*  (TERF1 interacting nuclear factor 2) |  |  |  |  | (Glousker et al., 2015; Milosic et al., 2024; OMIM) |
| *TERT*  (telomerase reverse transcriptase) |  |  |  |  | (Glousker et al., 2015; Milosic et al., 2024; OMIM) |
| *RTEL1*  (regulator of telomere elongation helicase 1) |  |  |  |  | (Carrero et al., 2016; Schnabel et al., 2021; Glousker et al., 2015; Milosic et al., 2024; OMIM) |
| *ACD*  (ACD shelterin complex subunit and telomerase recruitment factor)  alias symbol *TPP1* |  |  |  |  | (Carrero et al., 2016; Schnabel et al., 2021; Glousker et al., 2015; Milosic et al., 2024) |
| *NBN*  (nibrin)  previous symbols *NBS* and *NBS1* | NBN  (Nijmegen breakage  syndrome) | / | growth; head & neck; respiratory; abdomen; genitourinary; skin, nails & hair; neurologic; endocrine features; hematology; immunology; neoplasia | / | (Carrero et al., 2016; Rieckher et al., 2021; Worm et al., 2024; Schnabel et al., 2021; Martin and Oshima, 2000; Milosic et al., 2024; OMIM) |
| *POLR3A*  (RNA polymerase III subunit A) | WDRTS  (Wiedemann-Rautenstrauch syndrome) = NPS (neonatal progeroid syndrome) | / | growth; head & neck; respiratory; chest; abdomen; genitourinary; skeletal; skin, nails & hair; muscle, soft tissues; neurologic; voice; endocrine features | / | (Coppedè, 2021; Worm et al., 2024; Schnabel et al., 2021; Lessel and Kubisch, 2019; Hennekam, 2020; Beauregard-Lacroix et al., 2020; OMIM) |
| *LEMD2*  (LEM domain nuclear envelope protein 2) | MARUPS  (Marbach-Rustard progeroid syndrome) | / | growth; head & neck; cardiovascular; chest; abdomen; skeletal; skin, nails & hair; muscle, soft tissues; neurologic; prenatal manifestations | / | (Worm et al., 2024; Marbach et al., 2019; Marcelot et al., 2021; OMIM) |
| *SPRTN*  (SprT-like N-terminal domain) | RJALS  (Ruijs-Aalfs syndrome) | / | growth; head & neck; chest; abdomen; skeletal; skin, nails & hair; muscle, soft tissues; neoplasia | / | (Coppedè, 2021; Lessel et al., 2014; Worm et al., 2024; Schnabel et al., 2021; Burla et al., 2018; Hisama et al., 2016; OMIM) |
| *SLC25A24*  (solute carrier family 25 member 24) | FPS  (Fontaine progeroid syndrome) | / | growth; head & neck; cardiovascular; respiratory; chest; abdomen; genitourinary; skeletal; skin, nails & hair; muscle, soft tissues; neurologic; prenatal manifestations | / | (Coppedè, 2021; Worm et al., 2024; Schnabel et al., 2021; Hennekam, 2020; Writzl et al., 2017; OMIM) |
| *FBN1*  (fibrillin 1) | MFLS  (Marfanoid-progeroid-lipodystrophy syndrome) | / | growth; head & neck; cardiovascular; chest; skeletal; skin, nails & hair; muscle, soft tissues; neurologic; prenatal manifestations | / | (Koschitzki et al., 2023; Coppedè, 2021; Schnabel et al., 2021; Hennekam, 2020; OMIM) |
| *PDGFB*  (platelet derived growth factor subunit B) | PENTT  (Penttinen-type premature aging syndrome) | / | growth; head & neck; skeletal; skin, nails & hair; muscle, soft tissues; neurologic | / | (Coppedè, 2021; Worm et al., 2024; Schnabel et al., 2021; Hennekam, 2020; OMIM) |
| *PIK3R1*  (phosphoinositide-3-kinase regulatory subunit 1) | SHORT syndrome  (short stature, hyperextensibility, hernia, ocular depression, Rieger anomaly, and teething delay syndrome) | / | growth; head & neck; genitourinary; skeletal; skin, nails & hair; muscle, soft tissues; neurologic; endocrine features | / | (Coppedè, 2021; Worm et al., 2024; Schnabel et al., 2021; Hennekam, 2020; OMIM) |
| *AGPAT2*  (1-acylglycerol-3-phosphate O-acyltransferase 2) | BSS/CGL  (Berardinelli-Seip syndrome / congenital generalized lipodystrophy) | CGL1  (CGL, type 1) | growth; head & neck; cardiovascular; abdomen; genitourinary; skeletal; skin, nails & hair; muscle, soft tissues; endocrine features | / | (Hisama et al., 2016; Pignolo et al., 2020; OMIM) |
| *BSCL2*  (BSCL2 lipid droplet biogenesis associated, seipin) |  | CGL2  (CGL, type 2) | growth; head & neck; cardiovascular; abdomen; genitourinary; skeletal; skin, nails & hair; muscle, soft tissues; neurologic; voice; endocrine features |  | (Hisama et al., 2016; Pignolo et al., 2020; OMIM) |
| *CAV1*  (caveolin 1) |  | CGL3  (CGL, type 3) | growth; head & neck; abdomen; skin, nails & hair; muscle, soft tissues; metabolic features; endocrine features |  | (Hisama et al., 2016; OMIM) |
| *CAVIN1*  (caveolae associated protein 1)  previous symbol *PTFR*; alias symbol *CGL4* |  | CGL4  (CGL, type 4) | growth; cardiovascular; abdomen; skeletal; skin, nails & hair; muscle, soft tissues; endocrine features; immunology |  | (Hisama et al., 2016; OMIM) |
| *PCYT1A*  (phosphate cytidylyltransferase 1A, choline) |  | CGL5  (CGL, type 5) | growth; abdomen; muscle, soft tissues; endocrine features |  | (OMIM; Payne et al., 2014) |
| *FBLN5*  (fibulin 5) | CL  (cutis laxa) | ARCL1A  (autosomal recessive CL, type IA) | growth; head & neck; cardiovascular; respiratory; chest; abdomen; genitourinary; skeletal; skin, nails & hair; prenatal manifestations | ARCL are autosomal recessive CL forms, ADCL are autosomal dominant CL forms. | (Koschitzki et al., 2023; Kariminejad et al., 2017; Driver et al., 2020; Verlee et al., 2021; Callewaert et al., 2011; OMIM) |
| *EFEMP2*  (EGF containing fibulin extracellular matrix protein 2)  alias symbol *FBLN4* |  | ARCL1B  (autosomal recessive CL, type IB) | growth; head & neck; cardiovascular; respiratory; chest; genitourinary; skeletal; skin, nails & hair; neurologic; prenatal manifestations |  | (Koschitzki et al., 2023; Kariminejad et al., 2017; Driver et al., 2020; Verlee et al., 2021; Callewaert et al., 2011; OMIM) |
| *LTBP4*  (latent transforming growth factor beta binding protein 4) |  | ARCL1C  (autosomal recessive CL, type IC) = Urban-Rifkin-Davis syndrome | growth; head & neck; cardiovascular; respiratory; chest; abdomen; genitourinary; skeletal; skin, nails & hair; muscle, soft tissues |  | (Koschitzki et al., 2023; Kariminejad et al., 2017; Verlee et al., 2021; Callewaert et al., 2011; Pottie et al., 2021; OMIM) |
| *EFEMP1*  (EGF containing fibulin extracellular matrix protein 1)  alias symbol *FBLN3* |  | ARCL1D  (autosomal recessive CL, type ID) | growth; head & neck; respiratory; chest; abdomen; genitourinary; skeletal; skin, nails & hair; muscle, soft tissues; neurologic |  | (OMIM; Driver et al., 2020; Verlee et al., 2021) |
| *ATP6V0A2*  (ATPase H+ transporting V0 subunit a2) |  | ARCL2A  (autosomal recessive CL, type IIA) | growth; head & neck; abdomen; skeletal; skin, nails & hair; muscle, soft tissues; neurologic |  | (Koschitzki et al., 2023; Schnabel et al., 2021; Kariminejad et al., 2017; Reversade et al., 2009; Kornak et al., 2008; Callewaert et al., 2011; OMIM) |
| *PYCR1*  (pyrroline-5-carboxylate reductase 1) |  | ARCL2B  (autosomal recessive CL, type IIB) | growth; head & neck; cardiovascular; abdomen; skeletal; skin, nails & hair; neurologic |  | (Coppedè, 2021; Schnabel et al., 2021; Reversade et al., 2009; Marbach et al., 2019; Kariminejad et al., 2017; Lessel and Kubisch, 2019; Callewaert et al., 2011; OMIM) |
| *PYCR1*  (pyrroline-5-carboxylate reductase 1) |  | ARCL3B  (autosomal recessive CL, type IIIB) = DE BARSY progeroid syndrome B | growth; head & neck; cardiovascular; genitourinary; skeletal; skin, nails & hair; neurologic |  | (Koschitzki et al., 2023; Coppedè, 2021; Schnabel et al., 2021; Reversade et al., 2009; Marbach et al., 2019; Kariminejad et al., 2017; Lessel and Kubisch, 2019; Callewaert et al., 2011; OMIM) |
| *ATP6V1E1*  (ATPase H+ transporting V1 subunit E1) |  | ARCL2C  (autosomal recessive CL, type IIC) | growth; head & neck; cardiovascular; respiratory; genitourinary; skeletal; skin, nails & hair; muscle, soft tissues; neurologic; metabolic features |  | (Schnabel et al., 2021; OMIM) |
| *ATP6V1A*  (ATPase H+ transporting V1 subunit A) |  | ARCL2D  (autosomal recessive CL, type IID) | growth; head & neck; cardiovascular; genitourinary; skeletal; skin, nails & hair; muscle, soft tissues; neurologic; metabolic features |  | (Schnabel et al., 2021; OMIM) |
| *LTBP1*  (latent transforming growth factor beta binding protein 1) |  | ARCL2E  (autosomal recessive CL, type IIE) | growth; head & neck; cardiovascular; chest; abdomen; skeletal; skin, nails & hair; neurologic |  | (OMIM; Pottie et al., 2021) |
| *ALDH18A1*  (aldehyde dehydrogenase 18 family member A1) |  | ARCL3A  (autosomal recessive CL, type IIIA) = DE BARSY progeroid syndrome A | growth; head & neck; chest; abdomen; genitourinary; skeletal; skin, nails & hair; muscle, soft tissues; neurologic |  | (Koschitzki et al., 2023; Schnabel et al., 2021; Kariminejad et al., 2017; Callewaert et al., 2011; OMIM) |
| *FBLN5*  (fibulin 5) |  | ADCL2  (autosomal dominant CL 2) | cardiovascular; abdomen; skeletal; skin, nails & hair | OMIM indicates a provisional relationship between *FBLN5* and ADCL2. | (OMIM; Callewaert et al., 2011) |
| *ALDH18A1*  (aldehyde dehydrogenase 18 family member A1) |  | ADCL3  (autosomal dominant CL 3) | growth; head & neck; cardiovascular; abdomen; genitourinary; skeletal; skin, nails & hair; neurologic; prenatal manifestations |  | (Hennekam, 2020; OMIM) |
| *ELN*  (elastin) |  | ADCL1  (autosomal dominant CL 1) | head & neck; cardiovascular; respiratory; genitourinary; skin, nails & hair |  | (OMIM; Callewaert et al., 2011) |
| *GORAB*  (golgin, RAB6 interacting)  previous symbol *SCYL1BP1*; alias symbol *NTKL-BP1* | GO  (geroderma osteodysplasticum) | / | head & neck; skeletal; skin, nails & hair; neurologic | GO is sometimes also classified among progeroid CL (cutis laxa) syndromes (Kariminejad et al., 2017; Schnabel et al., 2021), due to many clinical overlaps, which can also complicate accurate diagnosis. Similarly, due to many phenotypic similarities, it has been proposed that GO and WSS (wrinkly skin syndrome) represent the same disorder (Nanda et al., 2008); whereas other studies suggest that WSS and GO are distinct entities with overlapping features (Rajab et al., 2008; OMIM). | (Worm et al., 2024; Schnabel et al., 2021; Egerer et al., 2015; Reversade et al., 2009; Callewaert et al., 2011; Kariminejad et al., 2017; OMIM) |
| *ATP6V0A2*  (ATPase H+ transporting V0 subunit a2) | WSS  (wrinkly skin syndrome) | / | growth; head & neck; respiratory; chest; abdomen; genitourinary; skeletal; skin, nails & hair; muscle, soft tissues; neurologic; voice; prenatal manifestations | Pathogenic variants in the same gene (*ATP6V0A2*) in ARCL2A (autosomal recessive cutis laxa type IIA) suggest that WSS and some cases of ARCL2A may represent variable manifestations of the same genetic defect (Kornak et al., 2008; OMIM). Due to overlapping features, these two disorders have sometimes been regarded as one disorder with a variable spectrum of severity; other studies, however, highlight the important differentiating features between ARCL2 and WSS (Gupta and Phadke, 2006). | (Kornak et al., 2008; Kariminejad et al., 2017; Callewaert et al., 2011; OMIM) |
| *TFAP2A*  (transcription factor AP-2 alpha) | BOFS  (branchiooculofacial syndrome) | / | growth; head & neck; chest; genitourinary; skeletal; skin, nails & hair; neurologic; voice; immunology | / | (Worm et al., 2024; Milunsky et al., 2008; OMIM) |
| *H1-4*  (H1.4 linker histone, cluster member)  previous symbol *HIST1H1E* | RMNS  (Rahman syndrome) | / | growth; head & neck; abdomen; skeletal; skin, nails & hair; muscle, soft tissues; neurologic | / | (Schnabel et al., 2021; Worm et al., 2024; OMIM) |
| *TREX1*  (three prime repair exonuclease 1) | AGS  (Aicardi-Goutières syndrome) | AGS1  (AGS 1) | head & neck; abdomen; skin, nails & hair; neurologic; hematology; immunology | / | (Hisama et al., 2016; Rice et al., 2012; Amari et al., 2020; Uggenti et al., 2020; OMIM) |
| *RNASEH2B*  (ribonuclease H2 subunit B) |  | AGS2  (AGS 2) | head & neck; neurologic |  | (Hisama et al., 2016; Rice et al., 2012; Amari et al., 2020; Uggenti et al., 2020; OMIM) |
| *RNASEH2C*  (ribonuclease H2 subunit C) |  | AGS3  (AGS 3) | head & neck; skin, nails & hair; muscle, soft tissues; neurologic |  | (Hisama et al., 2016; Rice et al., 2012; Amari et al., 2020; Uggenti et al., 2020; OMIM) |
| *RNASEH2A*  (ribonuclease H2 subunit A) |  | AGS4  (AGS 4) | growth; head & neck; respiratory; abdomen; neurologic; hematology; immunology |  | (Hisama et al., 2016; Rice et al., 2012; Amari et al., 2020; Uggenti et al., 2020; OMIM) |
| *SAMHD1*  (SAM and HD domain containing deoxynucleoside triphosphate triphosphohydrolase 1) |  | AGS5  (AGS 5) | head & neck; abdomen; skeletal; skin, nails & hair; neurologic; hematology |  | (Hisama et al., 2016; Rice et al., 2012; Amari et al., 2020; Uggenti et al., 2020; OMIM) |
| *ADAR*  (adenosine deaminase RNA specific)  alias symbol *ADAR1* |  | AGS6  (AGS 6) | head & neck; neurologic; hematology |  | (OMIM; Rice et al., 2012; Amari et al., 2020; Uggenti et al., 2020) |
| *IFIH1*  (interferon induced with helicase C domain 1)  alias symbol *MDA5* |  | AGS7  (AGS 7) | growth; head & neck; abdomen; genitourinary; skin, nails & hair; muscle, soft tissues; neurologic; hematology; immunology |  | (OMIM; Amari et al., 2020; Uggenti et al., 2020) |
| *LSM11*  (LSM11, U7 small nuclear RNA associated) |  | AGS8  (AGS 8) | head & neck; genitourinary; neurologic | OMIM indicates a provisional relationship between *LSM11* and AGS8. | (OMIM; Uggenti et al., 2020) |
| *RNU7-1* (RNA, U7 small nuclear 1) |  | AGS9  (AGS 9) | growth; head & neck; cardiovascular; abdomen; genitourinary; muscle, soft tissues; neurologic; endocrine features; hematology | / | (OMIM; Uggenti et al., 2020) |
| *B4GALT7*  (beta-1,4-galactosyltransferase 7) | EDSSPD  (Ehlers-Danlos syndrome, spondylodysplastic type) | EDSSPD1  (EDSSPD 1) | growth; head & neck; chest; skeletal; skin, nails & hair; neurologic | The former preferred titles/symbols of EDSSPD1 and EDSSPD2 included the term “progeroid type”. EDSSPD1 was called “Ehlers-Danlos Syndrome, Progeroid Type, 1; EDSP1,” and EDSSPD2 was “Ehlers-Danlos Syndrome, Progeroid Type, 2; EDSP2,” (OMIM). | (OMIM; Miyake et al., 2014; Leoni et al., 2021) |
| *B3GALT6*  (beta-1,3-galactosyltransferase 6) |  | EDSSPD2  (EDSSPD 2) | growth; head & neck; chest; skeletal; skin, nails & hair; neurologic |  | (OMIM; Miyake et al., 2014; Leoni et al., 2021) |
| *SLC39A13*  (solute carrier family 39 member 13) |  | EDSSPD3  (EDSSPD 3) | growth; head & neck; skeletal; skin, nails & hair; muscle, soft tissues | / | (OMIM; Giunta et al., 2008; Leoni et al., 2021) |
| *KCNJ6*  (potassium inwardly rectifying channel subfamily J member 6) | KPLBS  (Keppen-Lubinsky syndrome) | / | growth; head & neck; skeletal; skin, nails & hair; muscle, soft tissues; neurologic | / | (Worm et al., 2024; Masotti et al., 2015; OMIM) |
| *ANTXR1*  (ANTXR cell adhesion molecule 1) | GAPOS  (GAPO syndrome) | / | growth; head & neck; cardiovascular; respiratory; chest; abdomen; skeletal; skin, nails & hair; neurologic; endocrine features | / | (Worm et al., 2024; Schnabel et al., 2021; Abdel-Hamid et al., 2019; OMIM) |
| *COG4*  (component of oligomeric golgi complex 4) | SWILS  (Saul-Wilson syndrome) | / | growth; head & neck; chest; skeletal; neurologic; hematology | / | (Worm et al., 2024; Ferreira et al., 2018; OMIM) |
| *PTDSS1*  (phosphatidylserine synthase 1) | LMHD  (Lenz-Majewski hyperostotic dwarfism) | / | growth; head & neck; chest; abdomen; genitourinary; skeletal; skin, nails & hair; neurologic | / | (Schnabel et al., 2021; Sohn and Balla, 2016; OMIM) |
| *DDR2*  (discoidin domain receptor tyrosine kinase 2) | WRCN  (Warburg-Cinotti syndrome) | / | head & neck; cardiovascular; respiratory; skeletal; skin, nails & hair; muscle, soft tissues; endocrine features | / | (Hennekam, 2020; Xu et al., 2018; OMIM) |
| *TOR1AIP1*  (torsin 1A interacting protein 1) | LESSEL syndrome | / | NA | / | (Hennekam, 2020; Lessel et al., 2020; Mackels et al., 2023) |
| *DMPK*  (DM1 protein kinase) | DM  (myotonic dystrophy) | DM1  (DM 1) | head & neck; cardiovascular; abdomen; genitourinary; skin, nails & hair; muscle, soft tissues; neurologic; prenatal manifestations | / | (Koschitzki et al., 2023; Hisama et al., 2016; Lessel and Kubisch, 2019; Martin and Oshima, 2000; OMIM) |
| *CNBP*  (CCHC-type zinc finger nucleic acid binding protein) |  | DM2  (DM 2) | head & neck; cardiovascular; genitourinary; skin, nails & hair; muscle, soft tissues; neurologic; endocrine features; immunology |  | (Hisama et al., 2016; Martin and Oshima, 2000; OMIM) |
| *BUB1B*  (BUB1 mitotic checkpoint serine/threonine kinase B) | MVA  (mosaic variegated aneuploidy syndrome) | MVA1  (MVA 1) | growth; head & neck; chest; abdomen; genitourinary; neurologic; immunology; neoplasia; prenatal manifestations | / | (Kubben and Misteli, 2017; Yost et al., 2017; de Wolf et al., 2021; Grange et al., 2022; OMIM) |
| *CEP57*  (centrosomal protein 57) |  | MVA2  (MVA 2) | growth; head & neck; cardiovascular; respiratory; abdomen; skeletal; skin, nails & hair; neurologic; endocrine features | / | (Kubben and Misteli, 2017; Yost et al., 2017; de Wolf et al., 2021; Grange et al., 2022; OMIM) |
| *TRIP13*  (thyroid hormone receptor interactor 13) |  | MVA3  (MVA 3) | growth; head & neck; skin, nails & hair; neurologic; neoplasia | / | (OMIM; Yost et al., 2017; de Wolf et al., 2021; Grange et al., 2022) |
| *CENATAC*  (centrosomal AT-AC splicing factor)  previous symbol *CCDC84* |  | MVA4  (MVA 4) | head & neck; neurologic | OMIM indicates a provisional relationship between *CENATAC* and MVA4. | (OMIM; de Wolf et al., 2021) |
| *SLF2*  (SMC5-SMC6 complex localization factor 2) |  | MVA5  (MVA 5) = ATELS1 (Atelis syndrome 1) | growth; head & neck; cardiovascular; abdomen; skeletal; skin, nails & hair; neurologic; hematology; immunology | / | (OMIM; Grange et al., 2022) |
| *SMC5*  (structural maintenance of chromosomes 5) |  | MVA6  (MVA 6) = ATELS2 (Atelis syndrome 2) | growth; head & neck; cardiovascular; skeletal; skin, nails & hair; neurologic; hematology; immunology; neoplasia | / | (OMIM; Grange et al., 2022) |
| *RECQL*  (RecQ like helicase)  alias symbol *RECQL1* | RECON progeroid syndrome | / | growth; head & neck; skeletal; skin, nails & hair; muscle, soft tissues | / | (OMIM; Abu-Libdeh et al., 2022) |
| *TOMM7*  (translocase of outer mitochondrial membrane 7) | GMPGS  (Garg-Mishra progeroid syndrome) | / | growth; head & neck; respiratory; chest; abdomen; skeletal; skin, nails & hair; muscle, soft tissues; neurologic | / | (OMIM; Garg et al., 2022; Young et al., 2022) |
| *MDM2*  (MDM2 proto-oncogene) | LSKB  (Lessel-Kubisch syndrome) | / | growth; head & neck; cardiovascular; genitourinary; skin, nails & hair; voice | OMIM indicates a provisional relationship between *MDM2* and LSKB. | (Lessel et al., 2017; OMIM) |
| *POLR3GL*  (RNA polymerase III subunit GL) | SOFM  (short stature, oligodontia, dysmorphic facies, and motor delay) | / | growth; head & neck; cardiovascular; chest; abdomen; genitourinary; skeletal; muscle, soft tissues; neurologic | SOFM has also been described as a variant of neonatal progeroid syndrome, or WRS (Wiedemann-Rautenstrauch syndrome) (Beauregard-Lacroix et al., 2020). | (Beauregard-Lacroix et al., 2020; OMIM) |
| *TOP3A*  (DNA topoisomerase III alpha) | MGRISCE2  (microcephaly, growth restriction, and increased sister chromatid exchange 2) | / | growth; head & neck; cardiovascular; abdomen; skin, nails & hair; muscle, soft tissues; neurologic; immunology | MGRISCE2 has also been described as a BS (Bloom syndrome)-like disorder (Martin et al., 2018). | (Martin et al., 2018; OMIM) |
| *EGFR*  (epidermal growth factor receptor) | NNCIS  (neonatal nephrocutaneous inflammatory syndrome) | / | growth; head & neck; cardiovascular; respiratory; abdomen; genitourinary; skin, nails & hair; muscle, soft tissues; immunology; prenatal manifestations | / | (OMIM; Ganetzky et al., 2015; Mazurova et al., 2020) |
| *YRDC*  (yrdC N6-threonylcarbamoyltransferase domain containing) | GAMOS10  (Galloway-Mowat syndrome 10) | / | head & neck; abdomen; genitourinary; skeletal; skin, nails & hair; muscle, soft tissues; neurologic; endocrine features | / | (Schmidt et al., 2021; OMIM) |
| *EXOSC2*  (exosome component 2) | SHRF  (short stature, hearing loss, retinitis pigmentosa, and distinctive facies) | / | growth; head & neck; cardiovascular; skeletal; skin, nails & hair; neurologic; endocrine features | / | (Di Donato et al., 2016; OMIM) |
| *ACVR1*  (activin A receptor type 1) | FOP  (fibrodysplasia ossificans progressiva) | / | head & neck; respiratory; skeletal; skin, nails & hair; muscle, soft tissues; neurologic | FOP has been proposed as a segmental progeroid syndrome by Pignolo et al. (2020) due to a constellation of features resembling accelerated aging. The authors suggest that viewing FOP as a segmental progeroid syndrome offers a unique perspective on potential mechanisms of normal aging and may help identify new therapeutic targets for FOP. | (Pignolo et al., 2020; OMIM) |

NA indicates that subtype classification and/or corresponding clinical feature groups data could not be assigned based on the available OMIM annotations at the time of curation.
