# Supplementary material for: A manually curated gene–phenotype catalogue for progeroid syndromes and premature aging
Source: Aging (Albany NY). 2026 Mar 30;18(1):234–60. doi: 10.18632/aging.206366 (PMC13285954; doi:10.18632/aging.206366)
Supplement: Supplementary Table 2 [file aging-18-1-206366-s002.docx]

**Supplementary Table 2. A list of 144 genes associated with premature aging syndromes.**

| **Gene symbol** | **Gene name** | **Genomic location** | **Cytoband** | **Ensembl ID** | **NCBI ID** | **HGNC ID** |
| --- | --- | --- | --- | --- | --- | --- |
| *B3GALT6* | beta-1,3-galactosyltransferase 6 | 1:1232237-1235041(1) | p36.33 | ENSG00000176022 | 126792 | 17978 |
| *MAD2L2* | mitotic arrest deficient 2 like 2 | 1:11657230-11691811(-1) | p36.22 | ENSG00000116670 | 10459 | 6764 |
| *YRDC* | yrdC N6-threonylcarbamoyltransferase domain containing | 1:37802945-37808208(-1) | p34.3 | ENSG00000196449 | 79693 | 28905 |
| *ZMPSTE24* | zinc metallopeptidase STE24 | 1:40258041-40294180(1) | p34.2 | ENSG00000084073 | 10269 | 12877 |
| *SLC25A24* | solute carrier family 25 member 24 | 1:108134043-108200849(-1) | p13.3 | [ENSG00000085491](http://www.ensembl.org/homo_sapiens/Gene/Summary?db=core;g=ENSG00000085491) | 29957 | 20662 |
| *DCLRE1B* | DNA cross-link repair 1B | 1:113904619-113914086(1) | p13.2 | ENSG00000118655 | 64858 | 17641 |
| *POLR3GL* | RNA polymerase III subunit GL | 1:145964690-145978848(1) | q21.1 | ENSG00000121851 | 84265 | 28466 |
| *ADAR* | adenosine deaminase RNA specific | 1:154581695-154628013(-1) | q21.3 | ENSG00000160710 | 103 | 225 |
| *LMNA* | lamin A/C | 1:156082572-156140081(1) | q22 | ENSG00000160789 | 4000 | 6636 |
| *DDR2* | discoidin domain receptor tyrosine kinase 2 | 1:162631373-162787405(1) | q23.3 | ENSG00000162733 | 4921 | 2731 |
| *GORAB* | golgin, RAB6 interacting | 1:170531819-170553834(1) | q24.2 | ENSG00000120370 | 92344 | 25676 |
| *TOR1AIP1* | torsin 1A interacting protein 1 | 1:179882042-179920077(1) | q25.2 | ENSG00000143337 | 26092 | 29456 |
| *UBE2T* | ubiquitin conjugating enzyme E2 T | 1:202331544-202341984(-1) | q32.1 | ENSG00000077152 | 29089 | 25009 |
| *SPRTN* | SprT-like N-terminal domain | 1:231337104-231355023(1) | q42.2 | ENSG00000010072 | 83932 | 25356 |
| *LTBP1* | latent transforming growth factor beta binding protein 1 | 2:32946953-33399509(1) | p22.3 | ENSG00000049323 | 4052 | 6714 |
| *CRIPT* | CXXC repeat containing interactor of PDZ3 domain | 2:46616416-46685037(1) | p21 | ENSG00000119878 | 9419 | 14312 |
| *EFEMP1* | EGF containing fibulin extracellular matrix protein 1 | 2:55865967-55924139(-1) | p16.1 | ENSG00000115380 | 2202 | 3218 |
| *FANCL* | FA complementation group L | 2:58159243-58241410(-1) | p16.1 | ENSG00000115392 | 55120 | 20748 |
| *ANTXR1* | ANTXR cell adhesion molecule 1 | 2:69013176-69249327(1) | p13.3 | ENSG00000169604 | 84168 | 21014 |
| *ANAPC1* | anaphase promoting complex subunit 1 | 2:111611639-111884690(-1) | q13 | ENSG00000153107 | 64682 | 19988 |
| *ERCC3* | ERCC excision repair 3, TFIIH core complex helicase subunit | 2:127257290-127294166(-1) | q14.3 | ENSG00000163161 | 2071 | 3435 |
| *ACVR1* | activin A receptor type 1 | 2:157736251-157876330(-1) | q24.1 | ENSG00000115170 | 90 | 171 |
| *IFIH1* | interferon induced with helicase C domain 1 | 2:162267074-162318684(-1) | q24.2 | ENSG00000115267 | 64135 | 18873 |
| *MTX2* | metaxin 2 | 2:176269395-176338025(1) | q31.1 | ENSG00000128654 | 10651 | 7506 |
| *FANCD2* | FA complementation group D2 | 3:10026370-10101932(1) | p25.3 | ENSG00000144554 | 2177 | 3585 |
| *XPC* | XPC complex subunit, DNA damage recognition and repair factor | 3:14145145-14178621(-1) | p25.1 | ENSG00000154767 | 7508 | 12816 |
| *ATRIP* | ATR interacting protein | 3:48446710-48467645(1) | p21.31 | ENSG00000164053 | 84126 | 33499 |
| *TREX1* | three prime repair exonuclease 1 | 3:48465811-48467645(1) | p21.31 | ENSG00000213689 | 11277 | 12269 |
| *TRAIP* | TRAF interacting protein | 3:49828601-49856574(-1) | p21.31 | ENSG00000183763 | 10293 | 30764 |
| *ATP6V1A* | ATPase H+ transporting V1 subunit A | 3:113746770-113812056(1) | q13.31 | ENSG00000114573 | 523 | 851 |
| *CNBP* | CCHC-type zinc finger nucleic acid binding protein | 3:129167827-129183922(-1) | q21.3 | ENSG00000169714 | 7555 | 13164 |
| *CEP63* | centrosomal protein 63 | 3:134485699-134587789(1) | q22.2 | ENSG00000182923 | 80254 | 25815 |
| *ATR* | ATR serine/threonine kinase | 3:142449007-142578733(-1) | q23 | ENSG00000175054 | 545 | 882 |
| *TERC* | telomerase RNA component | 3:169764063-169765158(-1) | q26.2 | ENSG00000270141 | 7012 | 11727 |
| *PCYT1A* | phosphate cytidylyltransferase 1A, choline | 3:196214222-196287957(-1) | q29 | ENSG00000161217 | 5130 | 8754 |
| *PLK4* | polo like kinase 4 | 4:127880893-127899224(1) | q28.1 | ENSG00000142731 | 10733 | 11397 |
| *TRIP13* | thyroid hormone receptor interactor 13 | 5:892884-919357(1) | p15.33 | ENSG00000071539 | 9319 | 12307 |
| *TERT* | telomerase reverse transcriptase | 5:1253147-1295068(-1) | p15.33 | ENSG00000164362 | 7015 | 11730 |
| *TARS1* | threonyl-tRNA synthetase 1 | 5:33440696-33468091(1) | p13.3 | ENSG00000113407 | 6897 | 11572 |
| *ERCC8* | ERCC excision repair 8, CSA ubiquitin ligase complex subunit | 5:60866454-60945073(-1) | q12.1 | ENSG00000049167 | 1161 | 3439 |
| *PIK3R1* | phosphoinositide-3-kinase regulatory subunit 1 | 5:68215740-68301821(1) | q13.1 | ENSG00000145675 | 5295 | 8979 |
| *LSM11* | LSM11, U7 small nuclear RNA associated | 5:157743712-157760709(1) | q33.3 | ENSG00000155858 | 134353 | 30860 |
| *B4GALT7* | beta-1,4-galactosyltransferase 7 | 5:177600132-177610330(1) | q35.3 | ENSG00000027847 | 11285 | 930 |
| *NHP2* | NHP2 ribonucleoprotein | 5:178149463-178153894(-1) | q35.3 | ENSG00000145912 | 55651 | 14377 |
| *TFAP2A* | transcription factor AP-2 alpha | 6:10393186-10419659(-1) | p24.3 | ENSG00000137203 | 7020 | 11742 |
| *H1-4* | H1.4 linker histone, cluster member | 6:26156329-26157115(1) | p22.2 | ENSG00000168298 | 3008 | 4718 |
| *LEMD2* | LEM domain nuclear envelope protein 2 | 6:33771202-33789130(-1) | p21.31 | ENSG00000161904 | 221496 | 21244 |
| *FANCE* | FA complementation group E | 6:35452338-35467104(1) | p21.31 | ENSG00000112039 | 2178 | 3586 |
| *POLH* | DNA polymerase eta | 6:43576185-43620523(1) | p21.1 | ENSG00000170734 | 5429 | 9181 |
| *GTF2H5* | general transcription factor IIH subunit 5 | 6:158168350-158199344(1) | q25.3 | ENSG00000272047 | 404672 | 21157 |
| *TOMM7* | translocase of outer mitochondrial membrane 7 | 7:22812628-22822849(-1) | p15.3 | ENSG00000196683 | 54543 | 21648 |
| *MPLKIP* | M-phase specific PLK1 interacting protein | 7:40126027-40134622(-1) | p14.1 | ENSG00000168303 | 136647 | 16002 |
| *EGFR* | epidermal growth factor receptor | 7:55019017-55211628(1) | p11.2 | ENSG00000146648 | 1956 | 3236 |
| *ELN* | elastin | 7:74027789-74069907(1) | q11.23 | ENSG00000049540 | 2006 | 3327 |
| *CAV1* | caveolin 1 | 7:116524994-116561179(1) | q31.2 | ENSG00000105974 | 857 | 1527 |
| *POT1* | protection of telomeres 1 | 7:124822386-124929983(-1) | q31.33 | ENSG00000128513 | 25913 | 17284 |
| *XRCC2* | X-ray repair cross complementing 2 | 7:152644776-152676193(-1) | q36.1 | ENSG00000196584 | 7516 | 12829 |
| *GTF2E2* | general transcription factor IIE subunit 2 | 8:30578318-30658236(-1) | p12 | ENSG00000197265 | 2961 | 4651 |
| *WRN* | WRN RecQ like helicase | 8:31033788-31176138(1) | p12 | ENSG00000165392 | 7486 | 12791 |
| *NBN* | nibrin | 8:89924515-90003228(-1) | q21.3 | ENSG00000104320 | 4683 | 7652 |
| *PTDSS1* | phosphatidylserine synthase 1 | 8:96261902-96336995(1) | q22.1 | ENSG00000156471 | 9791 | 9587 |
| *NSMCE2* | NSE2 (MMS21) homolog, SMC5-SMC6 complex SUMO ligase | 8:125091679-125367125(1) | q24.13 | ENSG00000156831 | 286053 | 26513 |
| *RECQL4* | RecQ like helicase 4 | 8:144511288-144517845(-1) | q24.3 | ENSG00000160957 | 9401 | 9949 |
| *FANCG* | FA complementation group G | 9:35073835-35080004(-1) | p13.3 | ENSG00000221829 | 2189 | 3588 |
| *SMC5* | structural maintenance of chromosomes 5 | 9:70258978-70354873(1) | q21.12 | ENSG00000198887 | 23137 | 20465 |
| *FANCC* | FA complementation group C | 9:95099054-95426796(-1) | q22.32 | ENSG00000158169 | 2176 | 3584 |
| *XPA* | XPA, DNA damage recognition and repair factor | 9:97674909-97697340(-1) | q22.33 | ENSG00000136936 | 7507 | 12814 |
| *CDK5RAP2* | CDK5 regulatory subunit associated protein 2 | 9:120388875-120580170(-1) | q33.2 | ENSG00000136861 | 55755 | 18672 |
| *EXOSC2* | exosome component 2 | 9:130693721-130707288(1) | q34.12 | ENSG00000130713 | 23404 | 17097 |
| *AGPAT2* | 1-acylglycerol-3-phosphate O-acyltransferase 2 | 9:136673143-136687457(-1) | q34.3 | ENSG00000169692 | 10555 | 325 |
| *ERCC6* | ERCC excision repair 6, chromatin remodeling factor | 10:49454168-49539538(-1) | q11.23 | ENSG00000225830 | 2074 | 3438 |
| *DNA2* | DNA replication helicase/nuclease 2 | 10:68414064-68472121(-1) | q21.3 | ENSG00000138346 | 1763 | 2939 |
| *POLR3A* | RNA polymerase III subunit A | 10:77953148-78029522(-1) | q22.3 | ENSG00000148606 | 11128 | 30074 |
| *ALDH18A1* | aldehyde dehydrogenase 18 family member A1 | 10:95605941-95656711(-1) | q24.1 | ENSG00000059573 | 5832 | 9722 |
| *SLF2* | SMC5-SMC6 complex localization factor 2 | 10:100912963-100965134(1) | q24.31 | ENSG00000119906 | 55719 | 17814 |
| *STN1* | STN1 subunit of CST complex | 10:103856806-103918332(-1) | q24.33 | ENSG00000107960 | 79991 | 26200 |
| *FANCF* | FA complementation group F | 11:22622533-22625823(-1) | p14.3 | ENSG00000183161 | 2188 | 3587 |
| *DDB2* | damage specific DNA binding protein 2 | 11:47214465-47239217(1) | p11.2 | ENSG00000134574 | 1643 | 2718 |
| *SLC39A13* | solute carrier family 39 member 13 | 11:47407132-47416496(1) | p11.2 | ENSG00000165915 | 91252 | 20859 |
| *BSCL2* | BSCL2 lipid droplet biogenesis associated, seipin | 11:62689289-62709845(-1) | q12.3 | ENSG00000168000 | 26580 | 15832 |
| *RNASEH2C* | ribonuclease H2 subunit C | 11:65714005-65720818(-1) | q13.1 | ENSG00000172922 | 84153 | 24116 |
| *EFEMP2* | EGF containing fibulin extracellular matrix protein 2 | 11:65866441-65873592(-1) | q13.1 | ENSG00000172638 | 30008 | 3219 |
| *BANF1* | BAF nuclear assembly factor 1 | 11:66002228-66004149(1) | q13.1 | ENSG00000175334 | 8815 | 17397 |
| *MRE11* | MRE11 homolog, double strand break repair nuclease | 11:94415570-94493885(-1) | q21 | ENSG00000020922 | 4361 | 7230 |
| *CEP57* | centrosomal protein 57 | 11:95789965-95832693(1) | q21 | ENSG00000166037 | 9702 | 30794 |
| *ATM* | ATM serine/threonine kinase | 11:108222804-108369102(1) | q22.3 | ENSG00000149311 | 472 | 795 |
| *CENATAC* | centrosomal AT-AC splicing factor | 11:118998138-119015793(1) | q23.3 | ENSG00000186166 | 338657 | 30460 |
| *RNU7-1* | RNA, U7 small nuclear 1 | 12:6943816-6943878(1) | p13.31 | ENSG00000238923 | 100147744 | 34033 |
| *RECQL* | RecQ like helicase | 12:21468910-21501669(-1) | p12.1 | ENSG00000004700 | 5965 | 9948 |
| *MARS1* | methionyl-tRNA synthetase 1 | 12:57475445-57517569(1) | q13.3 | ENSG00000166986 | 4141 | 6898 |
| *MDM2* | MDM2 proto-oncogene | 12:68808177-68845544(1) | q15 | ENSG00000135679 | 4193 | 6973 |
| *ATP6V0A2* | ATPase H+ transporting V0 subunit a2 | 12:123712353-123761755(1) | q24.31 | ENSG00000185344 | 23545 | 18481 |
| *CPAP** | centrosome assembly and centriole elongation protein | 13:24882279-24922889(-1) | q12.13 | ENSG00000151849 | 55835 | 17272 |
| *BRCA2* | BRCA2 DNA repair associated | 13:32315086-32400268(1) | q13.1 | ENSG00000139618 | 675 | 1101 |
| *RNASEH2B* | ribonuclease H2 subunit B | 13:50909747-51024120(1) | q14.3 | ENSG00000136104 | 79621 | 25671 |
| *ERCC5* | ERCC excision repair 5, endonuclease | 13:102845831-102875995(1) | q33.1 | ENSG00000134899 | 2073 | 3437 |
| *TINF2* | TERF1 interacting nuclear factor 2 | 14:24238286-24242663(-1) | q12 | ENSG00000092330 | 26277 | 11824 |
| *FANCM* | FA complementation group M | 14:45135930-45200890(1) | q21.2 | ENSG00000187790 | 57697 | 23168 |
| *NIN* | ninein | 14:50719763-50831162(-1) | q22.1 | ENSG00000100503 | 51199 | 14906 |
| *FBLN5* | fibulin 5 | 14:91869411-91947987(-1) | q32.12 | ENSG00000140092 | 10516 | 3602 |
| *NOP10* | NOP10 ribonucleoprotein | 15:34339159-34343180(-1) | q14 | ENSG00000182117 | 55505 | 14378 |
| *BUB1B* | BUB1 mitotic checkpoint serine/threonine kinase B | 15:40161023-40221123(1) | q15.1 | ENSG00000156970 | 701 | 1149 |
| *RAD51* | RAD51 recombinase | 15:40694774-40732340(1) | q15.1 | ENSG00000051180 | 5888 | 9817 |
| *FBN1* | fibrillin 1 | 15:48408313-48645721(-1) | q21.1 | ENSG00000166147 | 2200 | 3603 |
| *CEP152* | centrosomal protein 152 | 15:48712928-48811146(-1) | q21.1 | ENSG00000103995 | 22995 | 29298 |
| *FANCI* | FA complementation group I | 15:89243945-89317261(1) | q26.1 | ENSG00000140525 | 55215 | 25568 |
| *BLM* | BLM RecQ like helicase | 15:90717346-90816166(1) | q26.1 | ENSG00000197299 | 641 | 1058 |
| *SLX4* | SLX4 structure-specific endonuclease subunit | 16:3581181-3611606(-1) | p13.3 | ENSG00000188827 | 84464 | 23845 |
| *ERCC4* | ERCC excision repair 4, endonuclease catalytic subunit | 16:13920138-13952348(1) | p13.12 | ENSG00000175595 | 2072 | 3436 |
| *PARN* | poly(A)-specific ribonuclease | 16:14435700-14632728(-1) | p13.12 | ENSG00000140694 | 5073 | 8609 |
| *PALB2* | partner and localizer of BRCA2 | 16:23603160-23641321(-1) | p12.2 | ENSG00000083093 | 79728 | 26144 |
| *USB1* | U6 snRNA biogenesis phosphodiesterase 1 | 16:57999546-58021618(1) | q21 | ENSG00000103005 | 79650 | 25792 |
| *ACD* | ACD shelterin complex subunit and telomerase recruitment factor | 16:67657512-67660810(-1) | q22.1 | ENSG00000102977 | 65057 | 25070 |
| *AARS1* | alanyl-tRNA synthetase 1 | 16:70251983-70289707(-1) | q22.1 | ENSG00000090861 | 16 | 20 |
| *COG4* | component of oligomeric golgi complex 4 | 16:70480568-70523560(-1) | q22.1 | ENSG00000103051 | 25839 | 18620 |
| *RFWD3* | ring finger and WD repeat domain 3 | 16:74621399-74666877(-1) | q23.1 | ENSG00000168411 | 55159 | 25539 |
| *FANCA* | FA complementation group A | 16:89726683-89816977(-1) | q24.3 | ENSG00000187741 | 2175 | 3582 |
| *WRAP53* | WD repeat containing antisense to TP53 | 17:7686071-7703502(1) | p13.1 | ENSG00000141499 | 55135 | 25522 |
| *CTC1* | CST telomere replication complex component 1 | 17:8224815-8248058(-1) | p13.1 | ENSG00000178971 | 80169 | 26169 |
| *TOP3A* | DNA topoisomerase III alpha | 17:18271428-18315007(-1) | p11.2 | ENSG00000177302 | 7156 | 11992 |
| *CAVIN1* | caveolae associated protein 1 | 17:42402449-42423256(-1) | q21.2 | ENSG00000177469 | 284119 | 9688 |
| *BRCA1* | BRCA1 DNA repair associated | 17:43044295-43170245(-1) | q21.31 | ENSG00000012048 | 672 | 1100 |
| *RAD51C* | RAD51 paralog C | 17:58692573-58735611(1) | q22 | ENSG00000108384 | 5889 | 9820 |
| *BRIP1* | BRCA1 interacting helicase 1 | 17:61679139-61863559(-1) | q23.2 | ENSG00000136492 | 83990 | 20473 |
| *PYCR1* | pyrroline-5-carboxylate reductase 1 | 17:81932384-81942412(-1) | q25.3 | ENSG00000183010 | 5831 | 9721 |
| *TYMS* | thymidylate synthetase | 18:657653-673578(1) | p11.32 | ENSG00000176890 | 7298 | 12441 |
| *ENOSF1* | enolase superfamily member 1 | 18:670318-712662(-1) | p11.32 | ENSG00000132199 | 55556 | 30365 |
| *RBBP8* | RB binding protein 8, endonuclease | 18:22798261-23026488(1) | q11.2 | ENSG00000101773 | 5932 | 9891 |
| *RNASEH2A* | ribonuclease H2 subunit A | 19:12806584-12813640(1) | p13.13 | ENSG00000104889 | 10535 | 18518 |
| *LTBP4* | latent transforming growth factor beta binding protein 4 | 19:40592883-40629818(1) | q13.2 | ENSG00000090006 | 8425 | 6717 |
| *ERCC2* | ERCC excision repair 2, TFIIH core complex helicase subunit | 19:45349837-45370918(-1) | q13.32 | ENSG00000104884 | 2068 | 3434 |
| *ERCC1* | ERCC excision repair 1, endonuclease non-catalytic subunit | 19:45407334-45478828(-1) | q13.32 | ENSG00000012061 | 2067 | 3433 |
| *DMPK* | DM1 protein kinase | 19:45769709-45782552(-1) | q13.32 | ENSG00000104936 | 1760 | 2933 |
| *POLD1* | DNA polymerase delta 1, catalytic subunit | 19:50384204-50418018(1) | q13.33 | ENSG00000062822 | 5424 | 9175 |
| *PCNA* | proliferating cell nuclear antigen | 20:5114953-5126626(-1) | p12.3 | ENSG00000132646 | 5111 | 8729 |
| *SAMHD1* | SAM and HD domain containing deoxynucleoside triphosphate triphosphohydrolase 1 | 20:36890229-36951893(-1) | q11.23 | ENSG00000101347 | 25939 | 15925 |
| *RTEL1* | regulator of telomere elongation helicase 1 | 20:63657810-63696253(1) | q13.33 | ENSG00000258366 | 51750 | 15888 |
| *KCNJ6* | potassium inwardly rectifying channel subfamily J member 6 | 21:37607373-38121345(-1) | q22.13 | ENSG00000157542 | 3763 | 6267 |
| *PCNT* | pericentrin | 21:46324124-46445769(1) | q22.3 | ENSG00000160299 | 5116 | 16068 |
| *ATP6V1E1* | ATPase H+ transporting V1 subunit E1 | 22:17592136-17628749(-1) | q11.21 | ENSG00000131100 | 529 | 857 |
| *PDGFB* | platelet derived growth factor subunit B | 22:39223359-39244982(-1) | q13.1 | ENSG00000100311 | 5155 | 8800 |
| *FANCB* | FA complementation group B | X:14690388-14873255(-1) | p22.2 | ENSG00000181544 | 2187 | 3583 |
| *RNF113A* | ring finger protein 113A | X:119870475-119871733(-1) | q24 | ENSG00000125352 | 7737 | 12974 |
| *DKC1* | dyskerin pseudouridine synthase 1 | X:154762742-154777689(1) | q28 | ENSG00000130826 | 1736 | 2890 |

**CPAP* is the current HGNC-approved symbol (formerly *CENPJ*; Ensembl release 113 annotation).
