# Supplementary material for: A manually curated gene–phenotype catalogue for progeroid syndromes and premature aging
Source: Aging (Albany NY). 2026 Mar 30;18(1):234–60. doi: 10.18632/aging.206366 (PMC13285954; doi:10.18632/aging.206366)
Supplement: Supplementary Table 5 [file aging-18-1-206366-s005.docx]

**Supplementary Table 5. *LMNA*-associated diseases and their respective clinical feature groups.**

| **Disease** | **Clinical feature groups** |
| --- | --- |
| HGPS  (Hutchinson-Gilford progeria syndrome) | growth; head & neck; cardiovascular; chest; skeletal; skin, nails & hair; muscle, soft tissues; neurologic; metabolic features; hematology |
| APSs/AWS  (atypical progeroid syndromes / atypical Werner syndrome) | NA |
| RSDM2  (restrictive dermopathy 2) | growth; head & neck; respiratory; chest; abdomen; genitourinary; skeletal; skin, nails & hair; prenatal manifestations |
| MADA  (mandibuloacral dysplasia with type A lipodystrophy) | growth; head & neck; chest; skeletal; skin, nails & hair; muscle, soft tissues; endocrine features |
| FPLD2  (familial partial lipodystrophy, type 2 (Dunnigan type))  Note: variants in *LMNA* can also cause atypical lipodystrophic syndromes different from Dunnigan-type (Shin and Worman, 2021; Worman, 2012). | head & neck; cardiovascular; abdomen; genitourinary; skin, nails & hair; muscle, soft tissues; neurologic; endocrine features |
| CMT2B1  (Charcot-Marie-Tooth disease, axonal, type 2B1) | skeletal; neurologic |
| CMD1A  (cardiomyopathy, dilated, 1A) | cardiovascular; neurologic |
| CMD-HH  (cardiomyopathy, dilated, with hypogonadotropic hypogonadism) = Malouf syndrome | head & neck; cardiovascular; chest; genitourinary; skeletal; muscle, soft tissues; neurologic; endocrine features |
| HHS-SI  (heart-hand syndrome, Slovenian type) | cardiovascular; skeletal; muscle, soft tissues; neurologic |
| EDMD2/EDMD-AD  (Emery-Dreifuss muscular dystrophy 2, autosomal dominant)  Note: LGMD1B (Limb girdle muscular dystrophy type 1B) has also been categorized as a separate disease entity (Kang et al., 2018; Rankin and Ellard, 2006; Shin and Worman, 2021; Worman, 2012; Worman and Bonne, 2007); however, in the OMIM database it is now noted that: “Some cases of Emery-Dreifuss muscular dystrophy-2 were previously classified as a form of limb-girdle muscular dystrophy (type 1B; LGMD1B). LGMD1B was characterised as an autosomal dominant, slowly progressive limb-girdle muscular dystrophy with age-related atrioventricular cardiac conduction disturbances and the absence of early contractures. Straub et al. (2018), on behalf of the LGMD workshop study group, reclassified LGMD1B as EDMD2”. | head & neck; cardiovascular; chest; skeletal; muscle, soft tissues |
| EDMD3/EDMD-AR  (Emery-Dreifuss muscular dystrophy 3, autosomal recessive) | head & neck; cardiovascular; skeletal; muscle, soft tissues; neurologic |
| MDCL  (muscular dystrophy, congenital, LMNA-related) | growth; head & neck; cardiovascular; respiratory; skeletal; muscle, soft tissues; neurologic; prenatal manifestations |

NA indicates that clinical feature groups data could not be assigned based on the available OMIM annotations at the time of curation.
